# Molecular cobalt corrole complex for the heterogeneous electrocatalytic reduction of carbon dioxide

**DOI:** 10.1038/s41467-019-11868-5

**Published:** 2019-08-27

**Authors:** Sabrina Gonglach, Shounik Paul, Michael Haas, Felix Pillwein, Sreekumar S. Sreejith, Soumitra Barman, Ratnadip De, Stefan Müllegger, Philipp Gerschel, Ulf-Peter Apfel, Halime Coskun, Abdalaziz Aljabour, Philipp Stadler, Wolfgang Schöfberger, Soumyajit Roy

**Affiliations:** 1 Institute of Organic Chemistry, Johannes Kepler University Linz, Altenberger Straße 69, 4040 Linz, Austria; 2 Eco-Friendly Applied Materials Laboratory (EFAML), Materials Science Centre, Department of Chemical Sciences, Mohanpur Campus, Indian Institute of Science Education and Research, Kolkata, 741246 West Bengal India; 3 Eco-Friendly Applied Materials Laboratory (EFAML), College of Chemistry, Central China Normal University, 152 Luoyu Road, Wuhan, 430079 Hubei P. R. China; 4 Institute of Semiconductor and Solid State Physics, Johannes Kepler University Linz, Altenberger Straße 69, 4040 Linz, Austria; 5 Inorganic Chemistry I, Ruhr-Universität Bochum NC 3/74, Universitätsstraße 150, D-44801 Bochum, Germany; 6 Fraunhofer UMSICHT, Osterfelder Straße 3, 46047 Oberhausen, Germany; 7 Institute of Physical Chemistry and Linz Institute of Organic Solar Cells, Johannes Kepler University Linz, Altenberger Straße 69, 4040 Linz, Austria

**Keywords:** Heterogeneous catalysis, Organometallic chemistry, Electrocatalysis, Carbon capture and storage, Materials for energy and catalysis

## Abstract

Electrochemical conversion of CO_2_ to alcohols is one of the most challenging methods of conversion and storage of electrical energy in the form of high-energy fuels. The challenge lies in the catalyst design to enable its real-life implementation. Herein, we demonstrate the synthesis and characterization of a cobalt(III) triphenylphosphine corrole complex, which contains three polyethylene glycol residues attached at the *meso*-phenyl groups. Electron-donation and therefore reduction of the cobalt from cobalt(III) to cobalt(I) is accompanied by removal of the axial ligand, thus resulting in a square-planar cobalt(I) complex. The cobalt(I) as an electron-rich supernucleophilic d^8^-configurated metal centre, where two electrons occupy and fill up the antibonding d_z_^2^ orbital. This orbital possesses high affinity towards electrophiles, allowing for such electronically configurated metals reactions with carbon dioxide. Herein, we report the potential dependent heterogeneous electroreduction of CO_2_ to ethanol or methanol of an immobilized cobalt A_3_-corrole catalyst system. In moderately acidic aqueous medium (pH = 6.0), the cobalt corrole modified carbon paper electrode exhibits a Faradaic Efficiency (FE%) of 48 % towards ethanol production.

## Abstract (web summary)

Electrochemical conversion of carbon dioxide to ethanol is one of the most challenging energy conversion reactions. Here the authors show selective electroreduction of carbon dioxide to ethanol by using a functionalized cobalt A3-corrole catalyst immobilized on a carbon paper electrode.

## Introduction

Minimizing of the CO_2_ concentration in the atmosphere is one of the most important challenges in our time^1,2^. Therefore, the electrochemical reduction of CO_2_ to value added chemicals is a sustainable strategy to solve the growing energy crisis, which at the same time has the potential to mitigate environmental pollution. In the past years, the electrochemical reduction of CO_2_ has been studied by several research groups to produce valuable products, for example carbon monoxide, formic acid, methane, ethanol, or methanol^3–5^. Particularly the transformation of CO_2_ in high-density alcohols, especially methanol and ethanol, is a cherished goal for chemists and environmental engineers alike^6,7^. Such transformation of CO_2_ to alcohols coupled with the oxidation of water to oxygen^8^ is a promising strategy^9^. However, the low reactivity of carbon dioxide in water with its large energy barrier (∆*E* = −1366.8 kJ mol^−1^)^10^ and the competing hydrogen evolution reaction, impedes such transformation, which makes the development of catalysts for electrocatalytic CO_2_ reduction to ethanol in aqueous environment a big challenge^1,2,10^. The thermodynamic reduction potential for CO_2_ to methanol and ethanol is 0.03 and 0.09 V (vs. RHE), respectively which is kinetically disfavored. Hence, often CO_2_ reducing catalysts end up accruing lot of energy to be operational at a higher potential. In this regard the use of a molecular catalyst with earth abundant elements, (Fe, Mn, Co, Cu, and Ni), especially with a cobalt metal center^11,12^ is a viable alternative as it offers a high degree of tunability with product selectivity at a low overpotential. As early as in 1980s chemists have been successful in reducing CO_2_ to CO via electrochemical methods employing catalysts containing different metals like Co^13^, Ni^14^, Re^15–17^, etc. Recently electrochemical reduction of CO_2_ to ethanol has been studied in various ways^18,19^, by tuning the applied potential^20^, pH^21^, and nature of the electrolyte^22^ with an aim to control the product selectivity and increase the Faradaic efficiency (FE) as well as to understand the underlying mechanistic pathways. Metal surfaces^23,24^, oxides^25^, and alloys^26,27^ are the most explored examples which show good FE for CO_2_ reduction but lack selectivity^28,29^ and work at higher overpotentials, involving complex synthetic procedures^30,31^. Emergent materials like B and N co-doped nanodiamonds exhibit excellent FE (93 %) and selectivity for conversion to ethanol, but work at higher overpotentials^10^. Moreover such heteroatom-doped materials^32^ often require a sophisticated synthetic procedure like chemical vapor deposition, making it hard for large scale implementation^33^. We now compare and contrast state of the art catalysts for CO_2_ electro-reduction to ethanol, all of which work at higher overpotentials with shorter activity time and have lower FE as compared to the catalyst reported here (Supplementary Table 1). For instance, Cu(100) works at −0.97 V vs. RHE yielding ethanol with a FE% of 14.7^29^. Further, use of Cu based nano-particles in an ensemble fashion (trans-CuEn) showed a FE% of ethanol formation to be 17 at −0.86 V vs. RHE^34^. While, with tailoring of cubic Cu nanocrystals to an edge length of 44 nm, FE% of 80 was achieved for CO_2_ reduction but the FE% for Ethanol formation was as low as 3.7^35^. On the other hand, alloys like Cu_*x*_Zn show that both the selectivity and FE% of ethanol formation can be governed by tuning the Cu:Zn ratio in the catalyst with a maximum FE% of ethanol formation 29.1 and current density (−3.8 mA cm^−2^) obtained for Cu_4_Zn at −1.05 V vs. RHE^36^. Similarly, electrodeposited Cu–Ag alloy films (6% Ag) exhibit a FE% of ethanol formation to be 25 at −0.7 V vs. RHE with a relatively high current density of −300 mA cm^−2^ ^37^. Ethanol was also obtained by tuning the layer thickness of Cu(I)oxide catalysts with a FE% of 9–16 (current density −35 mA cm^−2^) at −0.99 V vs. RHE^38^, while Cu_2_O derived Cu films exhibit FE% of 11.8 at −0.88 V vs. RHE with a current density of −31.2 mA cm^−2^ ^39^. Materials like nanodiamonds on B and N co-doping shows a very good FE% of 93 and selectivity toward ethanol^10^.

With the aim to synthesize effective and stable electrocatalysts for CO_2_ reduction for the selective ethanol formation, we focus on a molecular Co-corrole catalyst. Metal corroles are structural similar to metal porphyrins with both the metal centers and ligands participating in multielectron redox processes and are promising candidates for efficient proton-coupled electron transfer^40–42^. These metal complexes stabilize radical intermediates thus providing an effective pathway to facilitate C–C step-up^43,44^. Cobalt and iron corroles have been previously found to be catalytically active for CO_2_ reduction to CO^12^.

We thus explore this class of catalysts for CO_2_ electroreduction by using cobalt triphenylphosphine 5,10,15-tris(2,3,5,6-tetrafluoro-4-(MeO-PEG(7))thiophenyl) corrole Co(PPh_3_) (TpFPC) (-S-PEG(7)-OMe)_3_ (“Co-corrole”) where all the three pentafluorophenyl-groups on the *meso-*positions 5, 10, 15 are modified at the *para-*positions by thiol bound PEG(7)-OMe moieties^45–47^. In the present work, we demonstrate the reduction of CO_2_ to alcohols, formaldehyde, carboxylates, and hydrogen with a Co-corrole modified carbon paper electrode working at a low overpotential (−0.8 V vs. RHE) and show the long-term activity of Co-corrole modified carbon paper electrodes, making it as robust as metallic copper electrocatalysts. The molecular cobalt(III)-corrole catalyst is a significant improvement in the matter of selective reduction of CO_2_ to value added chemicals.

## Results

### Synthesis and electrochemical characterization of the catalyst

**Fig. 1:**
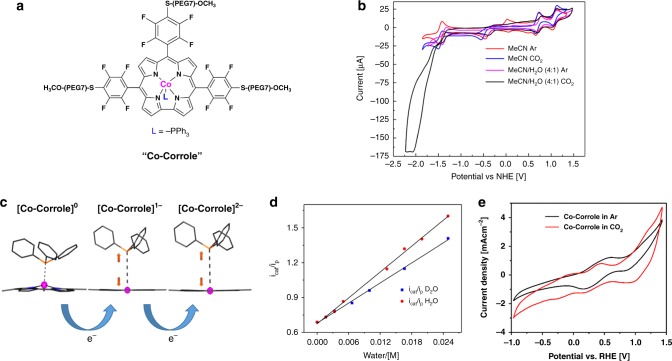
**a** Chemical structure of the Co-corrole. **b** Cyclic voltammetry of Co-corrole dissolved in CH_3_CN under Ar (red). Two metal centered redox peaks at −0.5 V (Co (III)/Co(II)) and −1.5 V (Co(II)/Co(I)) vs. NHE could be identified. The irreversibility of the redox peak at −0.5 V is likely due to the partial loss of the PPh_3_ ligand. Cyclic voltammetry of Co-corrole dissolved in CH_3_CN under CO_2_ (blue), in CH_3_CN/H_2_O (4:1) under Ar (pink) and in CH_3_CN/H_2_O (4:1) under CO_2_ (black). **c** DFT optimized geometries of [Co-corrole]^0^, 1e^−^ and 2e^−^ reduced species showing the movement of Co center into the central cavity of the corrole ring with concomitant lengthening of the Co-PPh_3_ bond upon successive reduction. **d** Kinetic isotpopic effect demonstrated by cyclic voltammetry of Co-Corrole (0.5 mM) recorded in CO_2_ saturated in acetonitrile in the presence of varying amount of H_2_O or D_2_O. Linear dependence of *i*_cat_/*i*_p_ on the concentration of water (analogous to plotting √k_CO2_ vs. [water], KIE = k_CO2H_/k_CO2D_ = (Slope_H2O_/slope_D2O_)^2^ = (37.1057/28.7545)^2^ = 1.67). All cyclic voltammograms were recorded with 0.1 M TBAPF_6_ as supporting electrolyte using a glassy carbon as working electrode and a Ag/AgCl as reference electrode at a scan rate of 100 mV s^−1^. **e** Heterogeneous catalysis of 1 mM Co-corrole on carbon-fiber electrode under Ar (black) and CO_2_ (red) at pH = 6.0 (Ag/AgCl/KCl, Pt, 0.1 M NaClO_4_, 100 mV s^−1^)

The Co-corrole was synthesized via four steps, where the first two steps leading to 5,10,15-trispentafluorophenylcorrole, were performed according to Gryko’s procedure^48^. The electron-withdrawing properties of the C_6_F_5_ functionalities render the corrole ring electron deficient^49^. Chemical modification with the -S-PEG(7)-OMe moieties at the three *para-*positions of the *meso*-C_6_F_5_ groups was performed (Fig. 1a) to optimize anchoring and equal distribution of the catalyst on the electrode surface. The chemical syntheses and characterizations are described in detail in the methods section and in the Supplementary Information file (Supplementary Figs. 1–6). The immobilization process of Co-corrole over carbon paper is implemented via drop casting by using anacetonitrile solution of Co-corrole. The modified carbon paper electrodes are stable in aqueous solution due to the insolubility of the Co-corrole moiety in water resulting in the formation of a sustainable heterogenized catalyst with extended life-time for electrocatalysis.

Electrochemical properties of the Co-corrole were investigated by cyclic voltammetry, with glassy carbon as working electrode in CH_3_CN under Argon and 0.1 M TBAPF_6_ as supporting electrolyte. As shown in Fig. 1b, red curve, two one electron redox peaks at −0.5 V (Co(III)/Co(II)) and −1.5 V (Co(II)/Co(I)) vs. NHE are occurring (Supplementary Fig. 7). The irreversibility of the redox peak at −0.5 V is likely due to the partial loss of the PPh_3_ ligand^50^. In analogy to previous report by Kadish et al.^50^, these two couples Co(III)/Co(II) and Co(II)/Co(I) are metal centered redox processes. The reversible two one electron redox peaks at 0.73 and 1.12 V vs. NHE are ligand centered oxidations and correspond to the formation of a cationic and dicationic cobalt(III) corrole complex^50^. DFT calculations suggest that the one-step and the two-step reduction at −0.5 and −1.5 V of the cobalt(III)-corrole leads to enhanced π-back bonding which strengthens the Co–N bonds and the cobalt corrole macrocycle becomes planar (Fig. 1c). Therefore, demetallation is energetically disfavored, which enables catalysis at a single Co(I)-site. The cyclic voltammetry of Co-corrole under CO_2_ (Fig. 1b, black curve) dissolved in CH_3_CN/H_2_O (4:1) shows compared with Co-corrole in ACN under CO_2_ (Fig. 1b, blue curve) a steep increase in current at −1.5 V vs. NHE, which confirms that CO_2_ reduction occurs in presence of a proton source. A value for the kinetic isotopic effect KIE = 1.64 was determined from the linear plots of *i*_cat_/*i*_p_ against [H_2_O] or [D_2_O] measured at 100 mV s^−1^ (Fig. 1d).

Cyclic voltammetry of the Co-corrole modified carbon–fiber electrode under Ar (Fig. 1e, black curve) and CO_2_ (Fig. 1e, red curve) was investigated in aqueous conditions at pH = 6.0, 7.2, and 8.0 (Supplementary Figs. 8–14, Co-corrole physisorbed on carbon paper). In all cases, a steep increase in catalytic current could be detected at a potential of −0.8 to −1.0 V vs. RHE where the second reduction reaction of [Co-corrole]^−^ to [Co-corrole]^2−^ occurs (Fig. 1e).

### Characterization of the electrocatalyst on the electrode surface

Cobalt triphenylphosphine 5,10,15–tris(2,3,5,6-tetrafluoro-4-(MeO-PEG(7))thiophenyl) corrole, Co(PPh_3_) (TpFPC) (-S-PEG(7)-OMe)_3_ (“Co-corrole”), is deposited on carbon paper (fabricated via drop casting using a acetonitrile mixture). The catalyst physisorbed on carbon paper was subjected to X-ray photoelectron spectroscopy (XPS) analysis before (Supplementary Table 2) and after the electroreduction (Supplementary Table 3). XPS survey scans exhibited the corresponding Co2p, Co3p, N1s, F1s, S2s, S2p, and P2p binding energy regions (Supplementary Fig. 15). The observed peaks correspond to Co2p, F1s, and P2p in the spectrum of Co-corroles as well as the absence of these peaks in the spectrum of non-modified carbon paper confirmed the presence of Co-corrole on the carbon paper. The high-resolution Co2p and N1s XP spectra (Supplementary Fig. 15) are in good agreement with previously published XPS data from CoTPP multilayer films^41,51^.

The main peak for Co 2p_3/2_ at 780.18 eV, is located at a typical cobalt(II) position (e.g., 780.2 eV for CoO) and the main peak for N1s is at 398.53 eV. Supplementary Fig. 15 displays also a C1s XP spectrum taken after electrocatalysis reaction. We observe two signals at 284.6 and 286.2 eV, because not all aromatic carbon atoms in the Co-corrole are the same, due to a lowering of symmetry to C_2v_ for corroles relative to D_4h_ for porphyrins. The shake-up satellite at 289.0 eV is typical for organic molecules with extended conjugated π systems. The XPS scans show that the catalyst is stable in course of electrocatalysis (Supplementary Fig. 15).

### Heterogeneous CO_2_ electroreduction

**Fig. 2:**
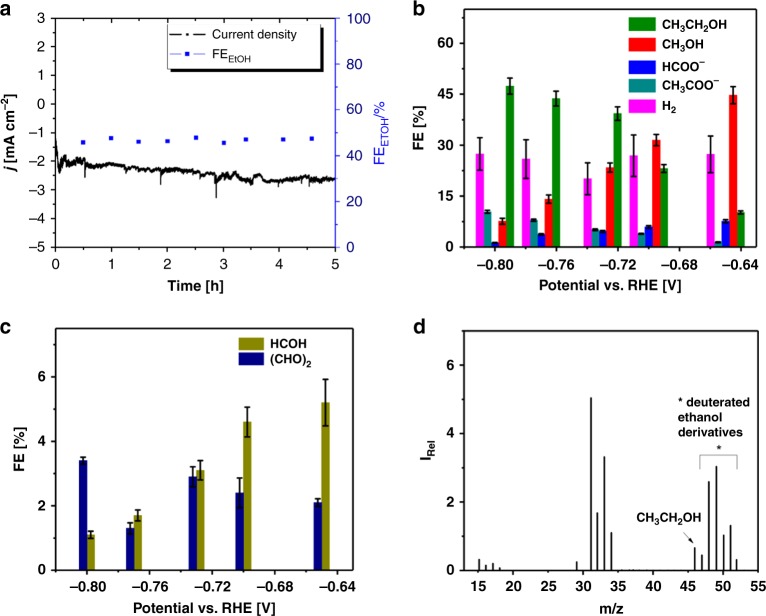
**a** Constant potential electrolysis of electrochemical CO_2_ reduction by the Co-corrole modified carbon paper electrode at a potential of −0.8 V vs. RHE (black curve), Faradaic efficiency for ethanol production over 5 h electrolysis (blue rectangles). **b** FE% vs. potential plot for potential dependent product formation. **c** FE% vs. potential plot for minor formed formaldehyde and solvated dimer of formaldehyde at different potentials. The error bars represent standard deviation of six measurements (three electrochemical reactions with two product analysis measurements for each reaction). **d** MS spectra obtained after electrolysis at −0.8 V vs. RHE in (1:3) D_2_O/H_2_O 0.1 M NaClO_4_ saturated with CO_2_

**Fig. 3:**
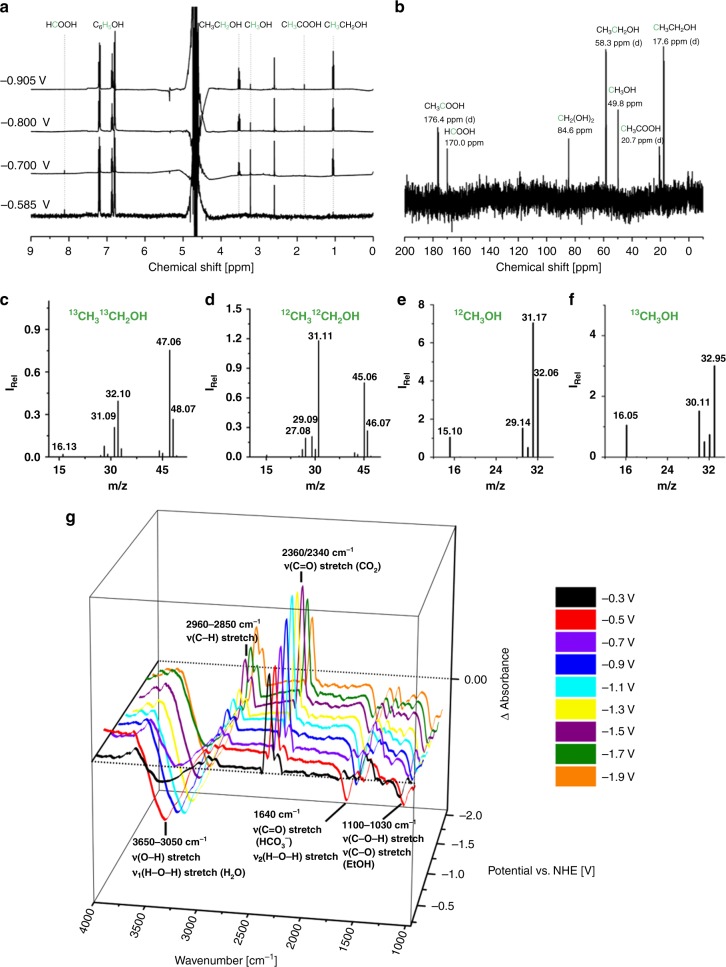
**a** ^1^H-NMR spectrum of the electrolyte after 5 h of CO_2_ electrolysis over Co-corrole–carbon paper electrode at −0.585, −0.7, −0.8, and −0.905 V vs. RHE in 0.1 M NaClO_4_, pH = 6, phosphate buffer, and phenol as the internal standard in DMSO. **b** ^13^C-NMR (101 MHz, H_2_O:D_2_O = 5:1) of the electrolyte after 5 h of electrolysis at −0.8 V vs. RHE in ^13^CO_2_, 0.1 M NaClO_4_, pH = 6, phosphate buffer. **c**–**f** GC–MS spectrum of the electrolyte after bulk electrolysis at −0.8 V vs. RHE in ^12^CO_2_ and ^13^CO_2_: **c** ^13^C enriched ethanol, **d** ^12^C enriched ethanol, **e** ^12^C enriched methanol, and **f** ^13^C enriched methanol. **g** SEC-FTIR spectra during the CO_2_ reduction with 20 mM Co-corrole in 0.1 M TBAPF_6_ (in CH_3_CN/H_2_O = 4:1), at potentials from −0.3 to −1.9 V vs. NHE

The heterogeneous electrochemical CO_2_ reduction experiments were carried out with Co-corrole deposited on carbon paper with effective loadings of 0.2 mg cm^−2^. The modified electrode was found to reduce CO_2_ to ethanol and methanol in 0.1 M NaClO_4_ at a potential of −0.8 V vs. RHE (pH = 6.0, 0.1 M phosphate buffer, Table 1). Controlled potential electrolysis (CPE) under CO_2_ of Co-corrole modified carbon paper exhibits a TON = 196 and a TOF = 0.011 s^−1^ for the catalytic conversion of CO_2_ to EtOH over 5 h (Fig. 2a–d). The quantification of products was performed using the observed ^1^H-nuclear magnetic resonance (NMR)- and gas chromatography mass spectrometry (GC–MS) measurement (e.g., in Fig. 3a and Table 1, and Supplementary Notes 1–3). XPS analysis of the electrode materials before and after catalysis reveals that the catalyst is stable in course of electroreduction and the catalyst pertaining to the reduction process are molecular Co-corrole units (Supplementary Fig. 15). Moreover, in the course of 5 h electrolysis at −0.8 V vs. RHE the Faradaic efficiency (FE%) for the ethanol production was measured at different intervals of time (Fig. 2a). Figure 2a illustrates that the FE = ~48% stays constant throughout the electrocatalytic reduction process. Subsequently, we have performed CPE with the modified carbon paper electrodes at different potentials, between −0.515 to −0.955 V vs. RHE for 5 h with current densities of 1.9–2.9 mA cm^−2^. H_2_ is the main gaseous compound and in the liquid phase ethanol, methanol, acetate, formaldehyde, glyoxal, and formate was detected. The quantifications of the respective gaseous and liquid compounds are summarized in Fig. 2b, c, Table 1, and Supplementary Figs. 16–23. During the CO_2_ reduction process, the FE% for ethanol was found in the range of 9–48 with the selectivity for C_2_ over C_1_ increasing with increase in the applied cathodic potential from −0.73 to −0.96 V vs RHE with a significant decrease in methanol production. CPE long-term measurements were performed at −0.73 V (pH = 6.0) and −0.70 V (pH = 7.2) vs. RHE, where the activity of the Co-corrole modified carbon electrodes retained for 140 h (Supplementary Figs. 8–13).

**Table 1:** Average Faradaic efficiency for each product detected on the Co-corrole-carbon paper electrode

Potential V vs. RHE	Faradaic Efficiency of each reduced products on Co-corrole-carbon paper electrode after 5 h of controlled potential electrolysis at the mentioned potentials^a^							Total FE%	Charge passed (Coulomb)
	CH_3_CH_2_OH	CH_3_OH	HCOO^−^	CH_3_COO^−^	H_2_	HCOH	(CHO)_2_		
	FE%	FE%	FE%	FE%	FE%	FE%	FE%		Avg.
−0.515	–	59	12	–	17	10	–	98	35
−0.585	5	52	10	–	22	6	3	98	36
−0.650	10	45	8	1	27	5	2	98	39
−0.700	23	32	6	4	27	5	2	99	40
−0.730	39	23	5	5	20	3	3	98	42
−0.770	44	14	4	8	26	2	1	99	43
−0.800	48	8	1	10	28	1	3	99	44
−0.855	47	5	–	12	33	–	2	99	47
−0.905	45	3	–	12	37	–	1	98	50
−0.955	47	2	–	13	36	–	–	98	53

^a^In 0.1 M NaClO_4_ (0.1 M pH = 6.0 phosphate buffer)

To further trace the origin of methanol and ethanol, we carried out CO_2_ reduction experiments with a D_2_O/H_2_O mixture. Deuterium distribution during the CO_2_ reduction process was monitored by conducting electrolysis at −0.8 V vs. RHE in D_2_O/H_2_O (1:3). After electrolysis, the mass spectra indicated the formation of reduced product and the following peaks appeared—*m*/*z* of 45–52 (Fig. 2d). Among the peaks obtained, the one with *m*/*z* value 52 (CD_3_CD_2_OD) can be assigned for hexa-deuterated ethanol. In the spectrum apart from the molecular ion peak, there are also peaks corresponding to other fragments with *m*/*z* = 50 which can be from CD_3_CD_2_O and *m*/*z* = 34 for CD_2_OD^+^ which are characteristic fragments of CD_3_CD_2_OD_._ This deuteration further proves that the source and incorporation of protons in the reduced product is from the solvent and the source of ethanol is from CO_2_ reduction. To confirm the source of carbon in the reduction products, we have conducted the reduction experiments with ^12^CO_2_ and ^13^CO_2_. The ^1^H and ^13^C-NMR spectra after CO_2_ reduction at the Co-corrole modified carbon paper electrode exhibit resonances for ethanol, methanol, acetic acid, and formic acid (Fig. 3a, b). The ^13^C-NMR spectrum after the reduction of ^13^CO_2_ clearly indicates four doublets at 58.3 ppm (^1^*J*_CC,EtOH_ = 37.5 Hz), 17.6 ppm (^1^*J*_CC,EtOH_ = 37.5 Hz), 176.4 ppm (^1^*J*_CC, acetic acid_ = 56.2 Hz), and 20.7 ppm (^1^*J*_CC, acetic acid_ = 56.2 Hz) indicating the reduction of ^13^CO_2_ and formation of C–C bond (Fig. 3b). HCOOH and CH_3_OH appear as singlets at 170.0 and 49.8 ppm in the ^13^C-NMR spectrum (Fig. 3b), respectively. These results were further substantiated by GC–MS data, which shows a shift of *m*/*z* = 2 for the molecular ion peak of CH_3_CH_2_OH^+^ on ^13^C enrichment (Fig. 3c, d). The results for CH_3_OH^+^, when compared to ^12^CO_2_ reduction shows a shift by *m*/*z* = 1 (Fig. 3e, f). On analysis of the fragmentation patterns, we observe that for ethanol obtained by the reduction of ^12^CO_2_, the peak at *m*/*z* = 29 corresponds to the CH_3_CH_2_^+^ ion. On the other hand, when ^13^CO_2_ is used, the peak occurs at *m*/*z* = 31, which is due to the substitution of both ^12^C centers with ^13^C isotopes. Likewise, the peak obtained at *m*/*z* = 31 in case of ^12^C enriched ethanol resembles the CH_2_OH^+^ fragment which on ^13^C enrichment shifts to *m*/*z* = 32. For ^12^C enriched methanol, the peak at *m*/*z* = 15 resembles CH_3_^+^ ion which shifts to *m*/*z* = 16 on ^13^C enrichment. The results obtained from both the ^13^C-NMR and GC–MS experiments prove that the source of ethanol as well as methanol is CO_2_ (Fig. 3b–f).

Spectroelectrochemical Fourier-transform infrared spectroscopy (FTIR) measurements (SEC-FTIR) at applied potentials of −0.3 to −1.9 V vs. NHE, illustrated in Fig. 3g exhibit increasing IR bands at 3400 cm^−1^ (O–H stretching), 2960 cm^−1^ (C–H stretching), 2850 cm^−1^ (C–H stretching), 1100–1060 cm^−1^ (C–O stretching), indicating the stepwise increase of [alcohol/acid] formation. The IR-band at 1640 cm^−1^ (C = O stretching of HCO_3_^−^) indicates the formation of a bicarbonate species. The corresponding IR bands for CO_2_ at 2360 and 2340 cm^−1^ (C = O stretching CO_2_) decrease during the reaction, indicating that CO_2_ is converted under these conditions.

## Discussion

An in-depth elucidation of the mechanistic pathway of the reduction process is beyond the scope of this present work. Detailed investigations are underway in our laboratories. To increase the CO_2_ reduction efficiency and to avoid hydrogen evolution at low-pH values, all experiments were performed under weak acidic conditions (pH = 6.0, 0.1 M phosphate buffer). CO_2_ reduction under heterogenized conditions with Co-corrole modified carbon electrodes exhibits a redox couple being [Co-corrole]^1−^/[Co-corrole]^2−^, which was found to be at −0.8 V vs. RHE. This markedly resembles the redox behavior of the Co-corrole molecule in the solution, so the redox properties are unperturbed upon heterogenization. We thus propose the molecular characteristics of the electrocatalyst to be persistent upon heterogenization. The EPR spectrum obtained after electrochemical reduction and subsequent dosage of CO_2_ at room temperature exhibits a rhombic *S* = 1/2 signal at *g* = 2 with a weak ^59^Co hyperfine splitting and indicates the formation of Co(III)–CO_2_^•−^ species (Supplementary Fig. 24).

The role of protons is extremely crucial in this work and sets this process apart from related CO_2_ reduction processes. For instance, at pH = 6.0 the necessary protons are provided for the subsequent proton coupled electron transfer (PCET), due to this, reduction to methanol/ethanol takes place (Fig. 3a). By performing the same experiment at pH = 7.2, we experimentally observe the reduction of CO_2_ to a mixture of formaldehyde, ethanol, methanol, acetate, and formate (see assignment of NMR resonances in the supplementary Fig. 20), and at a pH = 8.0 we detect only CO as the main reduction product (Supplementary Fig. 25a–c and Supplementary Notes 4, 5). This implies that the rate of PCET drastically decreases at weakly to moderately basic pH values. For the successful chemical transformation of CO_2_ to methanol and ethanol, this result is crucial in the present context.

The existence of the Co(III)–CO_2_^•−^ can only be justified through the presence of CO_2_^•−^ formed at a very high potential of *ca*. −1.5 V vs. RHE^52^. But in our case, the reduction wave at *ca*. −0.8 V vs. RHE is responsible for the CO_2_ reduction. This implies that the Co(I) in the center of the corrole complex enables the reduction of CO_2_ to the CO_2_^•−^ intermediate at a lower cost of energy.

**Fig. 4:**
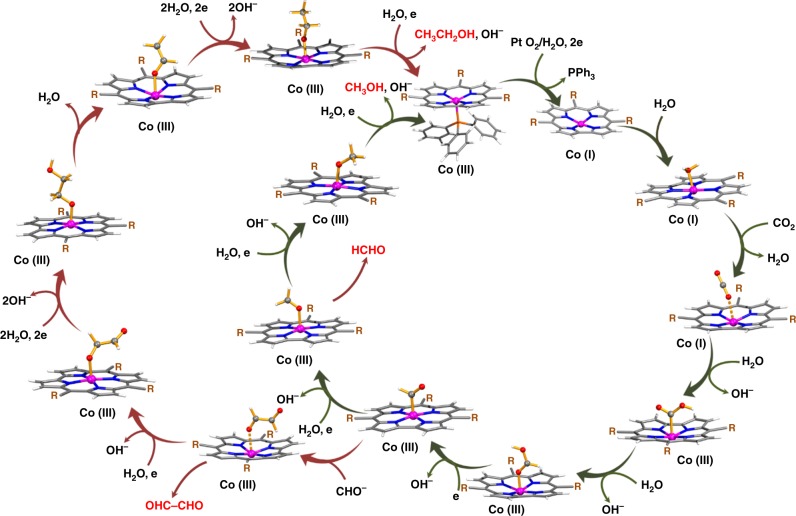
Proposed single site mechanism of CO_2_ reduction using Co-corrole

We suggest a mechanism similar to that proposed by Koper et al.^53^. which is illustrated in Fig. 4. The reaction follows a low energy pathway where the carboxyhydroxyl intermediate gets simultaneously reduced by 1 e^−^, gets protonated to form a HCO_2_H intermediate and can be detected by ^1^H-NMR spectroscopy (Fig. 3a, Supplementary Table 4, and Supplementary Notes 6–8). The HCO_2_H intermediate further undergoes 1e^−^ reduction with the elimination of OH^−^ to give HCO^•^ stabilized at the Co(III) site. Low potential for reactivity then drives the reaction toward methanol. This reduction pathway of HCO^•^ to CH_3_O^•^ at the cobalt site is in accordance with the mechanism reported by Koper et al. On the other hand, at a higher potential (>−0.73 V vs. RHE) an increased concentration of HCO^•^ is developed and_,_ the reaction takes a second route with the recombination of two formyl radicals (Co(III)–HCO^•^_+_ HCO^•^) leading to oxaldehyde intermediate (OHC–CHO). OHC–CHO was previously reported to be a precursor for the formation of ethanol as well as a key intermediate for C–C step-up leading to C_2_ products^54^. The π-conjugation present in OHC–CHO inherently makes it prone to reduction giving ^•^OCH_2_–CHO which subsequently reduces to ^•^OCH_2_–CH_2_OH which further reduces to CH_3_CH_2_OH. The high selectivity for alcohol is due to the formation of the HCO^•^ intermediate which is readily reducible to methanol. In the case of ethanol, we believe the formation of oxaldehyde intermediate opens up a more favorable route to form ethanol.

The proposed reaction pathways for the formation of the methanol and ethanol were further substantiated by conducting reduction studies of possible intermediates like formic acid, formaldehyde, methanol, and glyoxal under the same reaction conditions reported for Co-corrole–carbon paper electrodes. To test this conjecture, further reduction of the 2e^−^ reduced product formic acid was induced, where it yielded a mixture of methanol and ethanol which indeed implies formic acid to be an important intermediate. Cyclic voltammetric measurements show an increase in current density (Supplementary Fig. 34a) upon the addition of 10 mM of formic acid. CPE was performed at −0.73 V vs. RHE over Co-corrole–carbon paper for 5 h and the products obtained were analyzed in GC–MS spectrum where the presence of the characteristic peaks of methanol (*m*/*z* = 32) and ethanol (*m*/*z* = 46) were observed (Supplementary Figs. 36–38). The obtained results were further confirmed by ^1^H-NMR spectroscopy (Supplementary Fig. 34b). The proposed HCOOH intermediate under the low potential of reactivity gets reduced to HCHO and methanol, which are the final products and does not get further reduced. To verify the role of oxaldehyde in the current mechanistic cycle, 0.1 mM OHC–CHO was externally added under the reaction condition and CH_3_CH_2_OH was obtained proving oxaldehyde to be a key intermediate for CH_3_CH_2_OH formation (Supplementary Fig. 35a, b). The high selectivity for alcohol is primarily due to the formation of the formic acid intermediate followed by formation of HCO^•^ intermediate which is readily reducible to methanol. In the case of ethanol, we believe the formation of oxaldehyde is due to coupling of two HCO^•^ intermediate which opens up a more favorable route to form ethanol. To complete the studies, we performed comparative measurements with two similar Co-corroles (cobalt triphenylphosphine 5,10,15–tris(pentafluoro-phenyl) corrole (PPh_3_-CoTpFPC) and cobalt triphenylphosphine 5,10,15–triphenyl corrole PPh_3_-CoTPC)), which consist of (a) three *meso-* C_6_F_5_-groups or of (b) three *meso-* C_6_H_5_-groups. The results are illustrated in the Supplmentary Informations file of the paper (Supplementary Fig. 43). The main reduction products under the same reaction conditions at pH = 6 were assigned to formic acid, methanol, and acetic acid. Only trace amounts of ethanol were found employing these two catalyst systems.

To conclude, we have demonstrated the electrochemical reduction of CO_2_ to ethanol and methanol with a Co-corrole–carbon paper electrode at a low potential (−0.8 V vs. RHE) with a FE of 48% over a time period of 5 h. The Co-corrole–carbon paper electrode can withstand extremely high operational time of up to 140 h marking the highest efficiency for a molecular electrocatalyst reported so far in the literature. This is accomplished by the formation of a OHC–CHO type intermediate using a MeO-PEG(7)-S-modified cobalt corrole molecular catalyst. The Co-corrole molecule tends to stabilize different radical intermediates at the metal site. Thus, when reacted with a greater number of electrons highly reduced products are formed. On simulating the reactivity of Co-corrole, we found both experimentally and theoretically that in contrast to the CO pathway our catalyst proceeds via a formic acid pathway. By applying a redox potential of −0.73 V vs. RHE, a mixture of methanol and ethanol is detected. The obtained reaction products can only be explained if formic acid is developed temporarily, which is subsequently reduced to the formyl radical (HCO^•^). The HCO^•^ forms methanol as well as glyoxal and ultimately the glyoxal is then reduced to form ethanol serving our catalyst to operate at a low over potential.

## Methods

### Chemicals

All chemicals and solvents were commercially available and were used as obtained, unless otherwise noted. High-performance liquid chromatography grade solvents were used in all the experiments with water, methanol, acetonitrile, and sodium perchlorate (NaClO_4_) purchased from Merck, tetrabutylammonium perchlorate (Bu_4_NClO_4_), and Ferrocene from Sigma Aldrich. Tetrabutylammonium hexafluorophosphate (Bu_4_NPF_6_), 98%, purchased from Alfa Aesar, was twice recrystallized with absolute Ethanol, dried under high vacuum and stored under nitrogen. The 5,10,15-trispentafluorophenylcorrole was synthesized according to the Gryko’s method for highly reactive aldehydes^48^. For NMR experiments D_2_O was purchased from Cambridge Isotopic Laboratory. GC authentic standard ethanol, methanol, glyoxal, and diethoxymethane were purchased from Sigma Aldrich. Toray Carbon Paper, TGP-H-60, 19 × 19 cm was purchased from Alfa Aesar. 2,2,6,6-tetramethyl-1-piperidinyloxy, free radical, 2,2,6,6-tetramethylpiperidine 1-oxyl, TEMPO was purchased from Sigma Aldrich. ^13^CO_2_ (99.0 atom%) from Icon isotopes USA and Sigma Aldrich. CO_2_ and Ar used in the electrochemical measurements were purchased from Praxair with a purity of 99.995%.

### Materials synthesis and characterization

#### Free base 5,10,15-tris(2,3,5,6-tetrafluoro-4-(MeO-PEG(7))thiophenyl)corrole H_3_TpFPC(-S-PEG(7)-OMe)_3_

250 mg 5,10,15-tris(pentafluorophenyl)corrole (H_3_TpFPC) (314 µmol) and 225 mg sodium hydride (60% in mineral oil, 30 equiv., 9.4 mmol) were given in a 250 mL two-neck round-bottom flask under argon atmosphere. Totally, 100 mL THF_abs._ and 336 mg MeO-PEG(7)-thiol (3 equiv., 942 µmol) were added and the reaction was stirred at room temperature for 30 min. The reaction was controlled via TLC and mass spectroscopy. To quench the reaction, 15 mL H_2_O_dest._ were added. Further saturated aqueous NH_4_Cl-solution was added and the solution was extracted three times with ethylacetate. The combined organic phases were washed with 0.1 M HCl, dried over Na_2_SO_4_, filtered and evaporated to dryness. The product H_3_TpFPC(-S-PEG(7)-OMe)_3_ (517.8 mg, 0.267 mmol, 91.3 %) was obtained as a turquoise solid.

^**1**^**H-NMR** (300.1 MHz, CDCl_3_, 25 °C): *δ* = 9.09 (2H, d, ^3^*J* = 4.14 Hz, H2 + H18), 8.80 (2H, d, ^3^*J* = 4.38 Hz, H7 + H13), 8.61–8.58 (4H, m, H8 + H12 + H3 + H17), 3.91 (6H, t, ^3^*J* = 6.20 Hz, –CH_2_), 3.74–3.71 (12H, m, –CH_2_), 3.68–3.65 (12H, m, –CH_2_), 3.61 (12H, bs, –CH_2_), 3.56–3.55 (18H, m, –CH_2_), 3.52–3.49 (6H, m, –CH_2_), 3.42–3.39 (12H, m, –CH_2_), 3.26 (9H, s, –CH_3_) ppm (Supplementary Fig. 1).

^**19**^**F-NMR** (282.4 MHz, CDCl_3_, 25 °C): *δ* = −133.63 (4F, dd, ^3^*J* = 25.20 Hz, ^4^*J* = 11.87 Hz, Fo), −134.06 (2F, dd, ^3^*J* = 25.76 Hz, ^4^*J* = 12.12 Hz, Fo), −137.56 (2F, dd, ^3^*J* = 25.50 Hz, ^4^*J* = 12.21 Hz, Fm), −138.22 (4F, dd, ^3^*J* = 24.94 Hz, ^4^*J* = 11.73 Hz, Fm) ppm (Supplementary Fig. 2).

**HRMS** (ESI, positive): *m*/*z* calculated for C_82_H_104_F_12_N_4_O_21_S_3_: 1805.6236 [M+H]^+^, 1827.6061 [M + Na]^+^; found: 1805.6273 [M + H]^+^, 1827.6096 [M + Na]^+^ (Supplementary Fig. 3).

#### Co-Corrole: Co(PPh_3_) (TpFPC)(-S-PEG(7)-OMe)_3_

Totally, 385.2 mg of H_3_TpFPC(-S-PEG(7)-OMe)_3_ (0.213 mmol) were dissolved in 250 mL DCM: MeOH (2:1) in a 250 mL two-neck round-bottom flask under argon atmosphere. Cobalt(II)acetatetetrahydrate (539.7 mg, 2167 mmol, 10 equiv.) and triphenylphosphine (58.5 mg, 0.233 mmol, 1 equiv.) were added and the reaction mixture was heated to reflux for an hour. The reaction progress was monitored via UV/vis and TLC. After complete metalation, the mixture was extracted with DCM. The combined organic phases were extracted four times with water, dried over Na_2_SO_4_, filtered and evaporated under reduced pressure. Purification was performed with silica gel column chromatography (DCM/MeOH = 20:1), where the red product Co(PPh_3_)(TpFPC)(-S-PEG(7)-OMe)_3_ was obtained with 54.4% yield (246.3 mg, 115.9 µmol).

^**1**^**H-NMR** (300.1 MHz, CDCl_3_, 25 °C): *δ* = 8.68 (2H, d, ^3^*J* = 4.50 Hz, H2 + H18), 8.36 (2H, d, ^3^*J* = 4.77 Hz, H7 + H13), 8.26 (2H, d, ^3^*J* = 4.74 Hz, H8 + H12) 8.12 (2H, d, ^3^*J* = 4.47 Hz, H3 + H17), 7.04–6.99 (3H, m, triphenylphospine-CH), 6.69–6.64 (6H, m, triphenylphospine-CH), 4.64–4.58 (6H, m, triphenylphospine-CH), 3.91–3.86 (6H, m, –CH_2_), 3.74–3.69 (24 H, m, –CH_2_), 3.65–3.59 (42H, m, –CH_2_), 3.52–3.49 (6H, m, –CH_2_), 3.35 (9H, s, –CH_3_) ppm (Supplementary Fig. 4).

^**19**^**F-NMR** (282.4 MHz, CDCl_3_, 25 °C): *δ* = −133.88 to −133.72 (3F, m, Fo), −134.47 to −134.21 (3F, m, Fo), −136.99 (3F, dd, ^3^*J* = 25.47 Hz, ^4^*J* = 12.00 Hz, Fm), −137.30 (1F, dd, ^3^*J* = 25.44 Hz, ^4^*J* = 12.10 Hz, Fm), −138.08 (3F, dd, ^3^*J* = 25.81 Hz, ^4^*J* = 11.94 Hz, Fm) −138.44 (1F, dd, ^3^*J* = 25.80 Hz, ^4^*J* = 12.09 Hz, Fm) ppm (Supplementary Fig. 5).

**HRMS** (APPI, positive): *m*/*z* calculated for C_82_H_104_F_12_N_4_O_21_S_3_: 2145.6064 [M + Na]^+^; found: 2145.6078 [M + Na]^+^ (Supplementary Fig. 6).

### Preparation of the working electrode

The working electrode consists of cobalt(III)-corrole immobilized over carbon fiber paper (*A* = 1 cm^2^). For this, a 0.5 mmol solution of cobalt(III)-corrole in acetonitrile was prepared and drop-casted over carbon fiber to make a loading of 0.2 mg cm^−2^. Further, we preserve the electrodes at room temperature to remove the excess acetonitrile. Then these electrodes were washed with 0.1 M pH = 6 phosphate buffer to get rid of the acetonitrile completely. Then the electrodes were dried in presence of CaCl_2_ under high vacuum.

### Cyclic voltammetry and CPE

Cyclic Voltammetry and CPE for CO_2_ reduction were performed with a workstation (CH Instruments, Model CHI400A) in a two-compartment, three-electrode electrochemical H-cell, consisting of a gas tight cell with a total volume of 30 mL. Carbon paper was used as the working electrode. Ag/AgCl (*E*_Ag/AgCl_ = 0.222 V filled with 0.1 M KCl) as the reference, and Pt wire as the counter. The reference electrode potential was calibrated with respect to the reversible hydrogen potential using platinum working electrode and platinum wire as counter electrode in 0.5 M H_2_SO_4_ electrolyte in H_2_ atmosphere. This calibration result showed a shift of −0.222 V versus the NHE. All experiments were carried out at 25 °C. The pH value of the solutions were obtained using a EUTECH pH 510 pre-calibrated with Thermo Scientific pH 4.01, 7.0, and 10.01 buffer solutions. For all the measurements CO_2_ was continuously purged into the solution. All the potentials are represented in RHE scale with iR correction.

For electrochemistry in non-aqueous medium, acetonitrile was used as the solvent of choice with a similar electrochemical setup with glassy carbon as the working electrode, Ag/AgCl (0.1 M KCl) as the reference and Pt wire as the counter electrode with 0.1 M tetrabutylammonium perchlorate or 0.1 M tetrabutylammonium hexafluorophosphate as the electrolyte. Under this electrochemical conditions, the redox behavior of 0.01 M ferrocene in acetonitrile was studied, which was further used as the internal standard.

### Detection and quantification of the CO_2_ reduced products

^1^H- and ^13^C-NMR spectroscopy of the carbon dioxide reduced liquid products were recorded a on a Bruker Ascend 700 MHz Avance III NMR spectrometer equipped with a cryoprobe and on a JEOL ECS-400 NMR spectrometer. As internal standard, 20 mL aqueous solution of 20 mM phenol and 10 mM of dimethyl sulfoxide were used. After CPE, to 350 µL electrolyte, 200 µL D_2_O, and 50 µL of the internal standard were added and transferred into a NMR-tube. During the measurements, the water peak was suppressed to increase the signal intensity of the analytes. The CO_2_ reduced products were further analyzed using GC–MS. Trace 1300 GC and ISQ QD single quadruple GC–MS instrument with a TG-5MS capillary column (30 m × 0.32 mm × 0.25 µm) supplied by Thermo Fisher Scientific and DB-624 capillary column (30 m × 0.32 mm × 0.25 µm) supplied by Agilent were used for the same. For gaseous analysis CarboPLOT 007 capillary column (25 m × 0.53 mm × 0.25 µm) supplied by Agilent was used for separation and TCD for detection.

### Electrochemically active surface area (ECSA) calculation

ECSA value is obtained by using the following equation

1 $${\mathrm{ECSA}} = C_D/C_S$$

where *C*_*D*_ = electrochemical double layer capacitance which is obtained from the slope of the current vs. scan rate plot in the non-Faradaic region and *C*_*S*_ = specific capacitance of the sample and in this case, *C*_*S*_ = 0.040 mF cm^−2^ ^55^.

### Analysis of the CO_2_ reduced products in ^1^H-NMR and GC–MS

Compounds formed at different potentials were detected directly by ^1^H-NMR (integrated with respect to DMSO (*δ* = 2.71 ppm) as an internal standard) with a triplet signal at *δ* = 1.17 ppm and a quartet at *δ* = 3.67 ppm indicating ethanol (Supplementary Fig. 17); a singlet at *δ* = 3.37 ppm showing the presence of methanol (Supplementary Fig. 17).

For detection of formaldehyde, 2 mL of the reaction mixture was taken in a 20 ml headspace vial with 25 μL of ethanol and was acidified with 100 μL 1% *p*-toluenesulfonic acid. This mixture was then heated at 60 °C for 1 h and then GC–MS measurements were done^56^. In the mass spectrum, peak centered at *m*/*z* = 104 represents diethoxymethane indicating the formation of formaldehyde with a retention time around 3.00 min (Supplementary Figs. 39 and 40). Glyoxal was detected in the spectrum at *m*/*z* = 58 with a retention time of 3.35 min (Supplementary Figs. 41 and 42).

### XPS measurements

XPS was performed by using a Theta Probe, Thermofisher, UK, using monochromatic Al Kα X-rays (*hν* = 1486.6 eV), spot size 400 microns and with a photoelectron take-off angle of 90° with respect to the surface plane. The binding energies were corrected using the C1s peak at BE = 284.6 eV that arises from adventitious hydrocarbon.

## Supplementary information

Supplementary Information

## Data Availability

The data that support the findings of this study are available from the corresponding authors upon reasonable request.
