# Supplementary material for: Molecular cobalt corrole complex for the heterogeneous electrocatalytic reduction of carbon dioxide
Source: Nat Commun. 2019 Aug 27;10:3864. doi: 10.1038/s41467-019-11868-5 (PMC6711975; doi:10.1038/s41467-019-11868-5)
Supplement: Supplementary file 1 — Supplementary Information [file 41467_2019_11868_MOESM1_ESM.pdf]

Supplementary Information

Molecular cobalt corrole complex for the heterogeneous electrocatalytic reduction of carbon dioxide

Gonglach et al.

Supplementary Table 1. Benchmarking of well-known electrocatalysts reducing CO_2 to ethanol.

Sl no	Catalysts	Potential (V) / condition	Current density (mA cm^{-2})	FE (%) for ethanol	Other Products	Electrolyte	Reference
1	Cu(100)	-0.97 V vs RHE	-3.8	14.7	CH_4, C_2H_4 , hydrocarbons and C_2-C_3 products	0.1 M $KHCO_3$	**1**
2	Cu_4Zn	-1.05 V vs RHE	-8.2	29.1	C_2H_4	0.1 M $KHCO_3$	**2**
3	Trans-CuEn	-0.86 V vs RHE	-20.4	16.6	C_2-C_3 products, C_2H_4, *n*-propanol	0.1 M $KHCO_3$	**3**
4	B & N co-doped nanodiamonds	-1.0 V vs RHE.	-1.0 (approx)	93.2	No other products	0.1 M $NaHCO_3$	**4**
5	Cu_2O (1 mg cm^{-2})	−1.39 V vs. Ag/AgCl	-10	10.1	CH_3OH, C_3H_7OH	0.5 M $KHCO_3$	**5**
6	Cu_2O derived Cu catalyst	-0.88 V vs RHE.	-31.2	11.8	C_2H_4, *n*-propanaol	0.1 M $KHCO_3$	**6**
7	HKUST-1a (1 mg cm^{-2})	−0.9 V vs. Ag/AgCl	-10	10.3	CH_3OH	0.5 M $KHCO_3$	**7**
8	Cu nano crystals	-1.1 V vs RHE.	-9	3.7	C_2H_4, CH_3COOH, HCHO, $C_2H_4(OH)_2$, CH_3OH, C_3H_7OH	0.1 M $KHCO_3$	**8**
9	Cu(I) oxide catalyst	−0.99 V vs. RHE	-35	16	C_2H_4	0.1 M $KHCO_3$	**9**
10	Cu (3 10)	-1.42 V vs. SHE	−5	29.9	C_2H_4, CH_3COOH, HCHO, $C_2H_4(OH)_2$, CH_3OH, C_3H_7OH	0.1 M $KHCO_3$	**10**
11	Co Corrole	-0.8 V vs RHE	-2.5	48	CH_3OH	0.1 M $NaClO_4$ (pH = 6, 0.1 M phosphate buffer)	**This work**

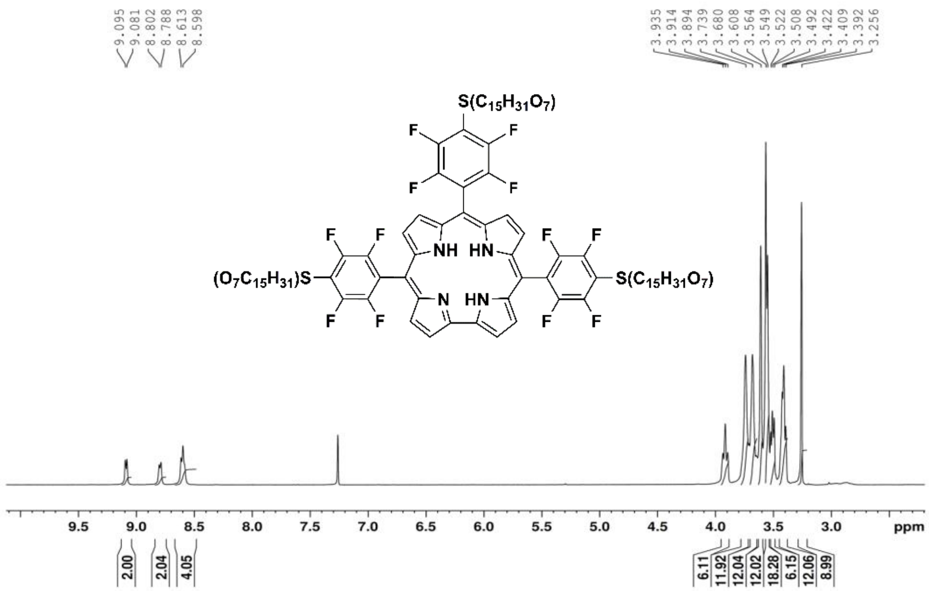

Supplementary Figure 1. ^{1}H-NMR spectrum of free base 5,10,15 – tris(2,3,5,6-tetrafluoro-4-(MeO-PEG(7))thiophenyl)corrole H_3TpFPC(-S-PEG(7)-OMe)$_3$.

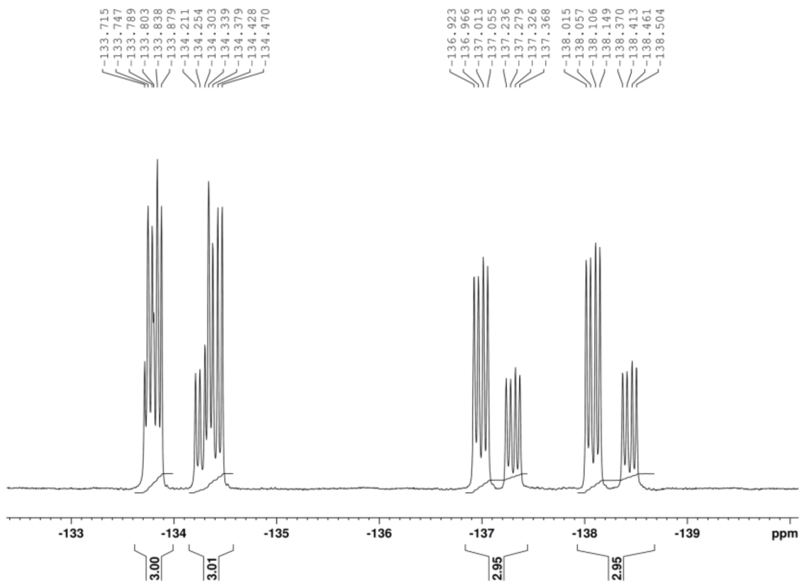

Supplementary Figure 2. ^{19}F-NMR spectrum of free base 5,10,15 – tris(2,3,5,6-tetrafluoro-4-(MeO-PEG(7))thiophenyl)corrole H_3TpFPC(-S-PEG(7)-OMe)$_3$.

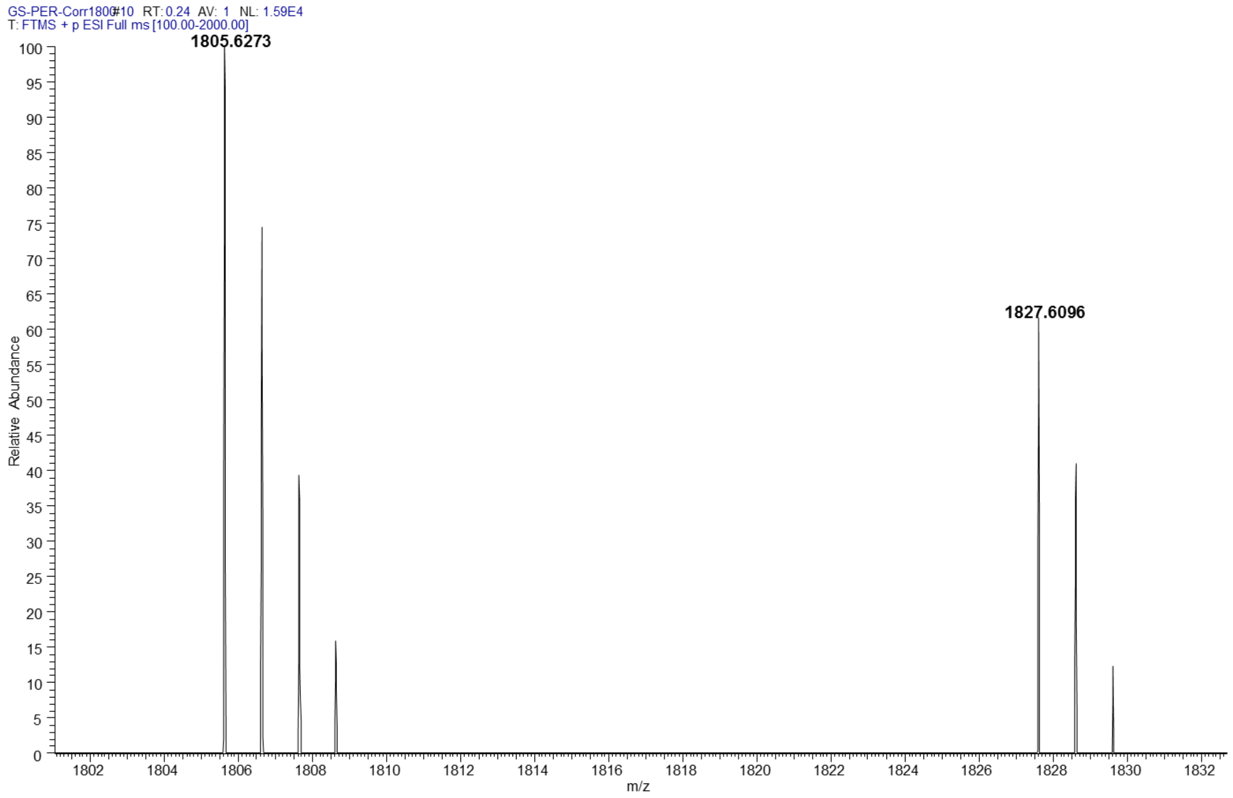

Supplementary Figure 3. HRMS of free base 5,10,15 – tris(2,3,5,6-tetrafluoro-4-(MeO-PEG(7))thiophenyl)corrole $H_3TpFPC(-S-PEG(7)-OMe)_3$.

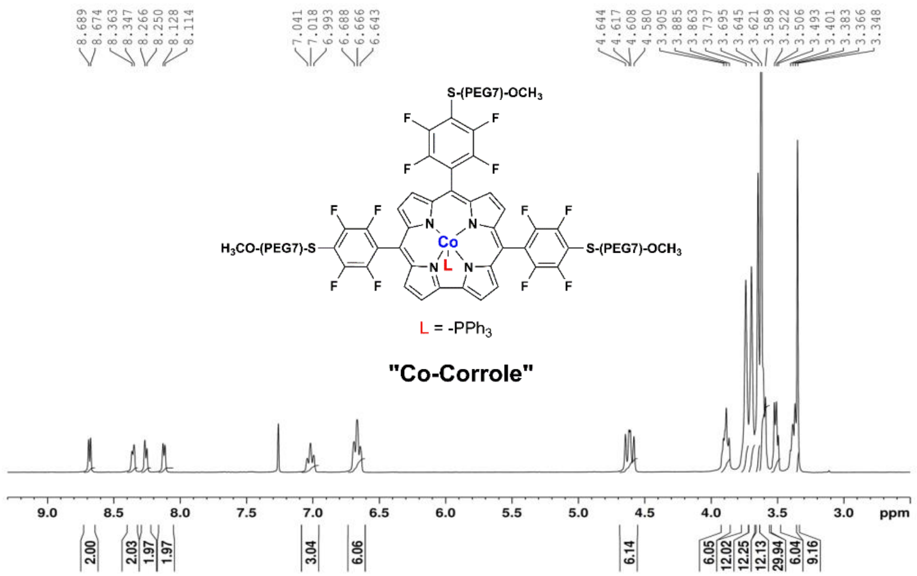

Supplementary Figure 4. ^{1}H-NMR spectrum of Cobalt triphenylphosphine 5,10,15 – tris(2,3,5,6-tetrafluoro-4-(MeO-PEG(7))thiophenyl)corrole Co(PPh$_3$) (TpFPC)(-S-PEG(7)-OMe)$_3$.

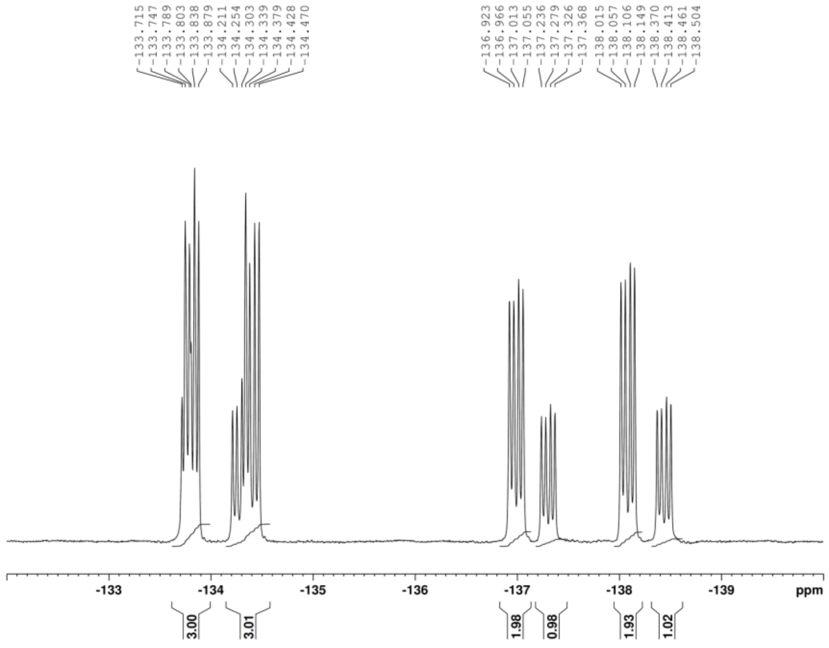

Supplementary Figure 5. ^{19}F-NMR spectrum of Cobalt triphenylphosphine 5,10,15 – tris(2,3,5,6-tetrafluoro-4-(MeO-PEG(7))thiophenyl)corrole Co(PPh$_3$) (TpFPC)(-S-PEG(7)-OMe)$_3$.

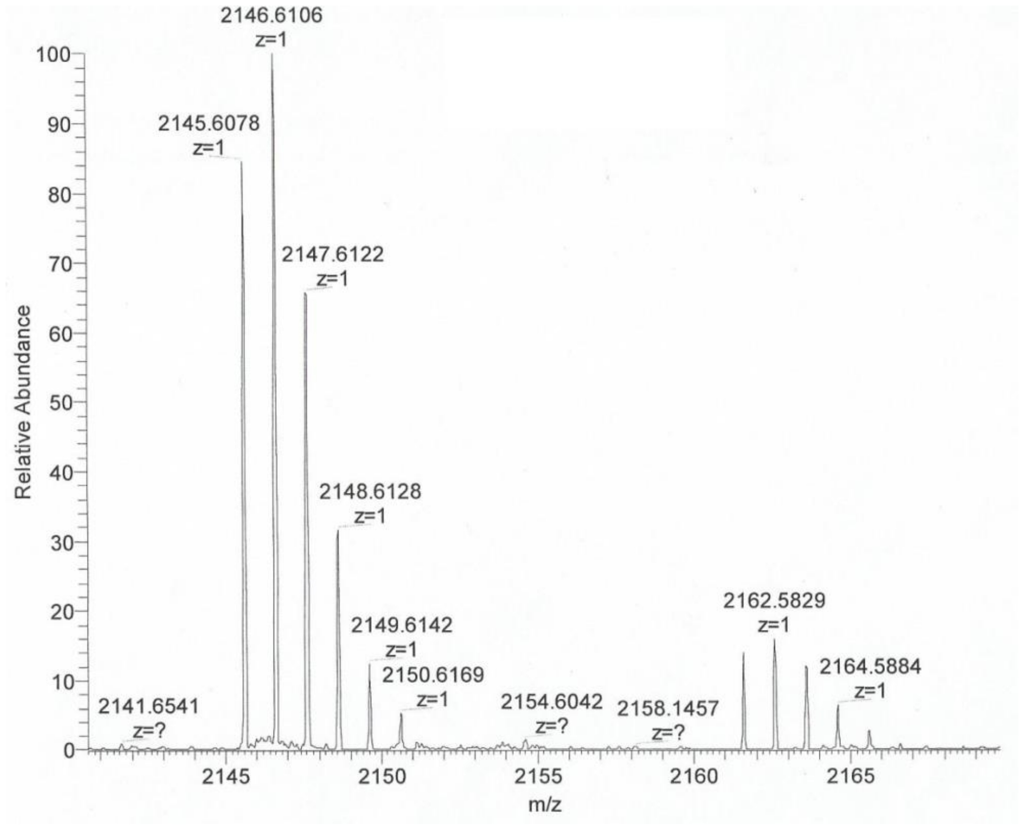

Supplementary Figure 6. HRMS of Cobalt triphenylphosphine 5,10,15 – tris(2,3,5,6-tetrafluoro-4-(MeO-PEG(7))thiophenyl)corrole Co(PPh_3) (TpFPC)(-S-PEG(7)-OMe)$_3$.

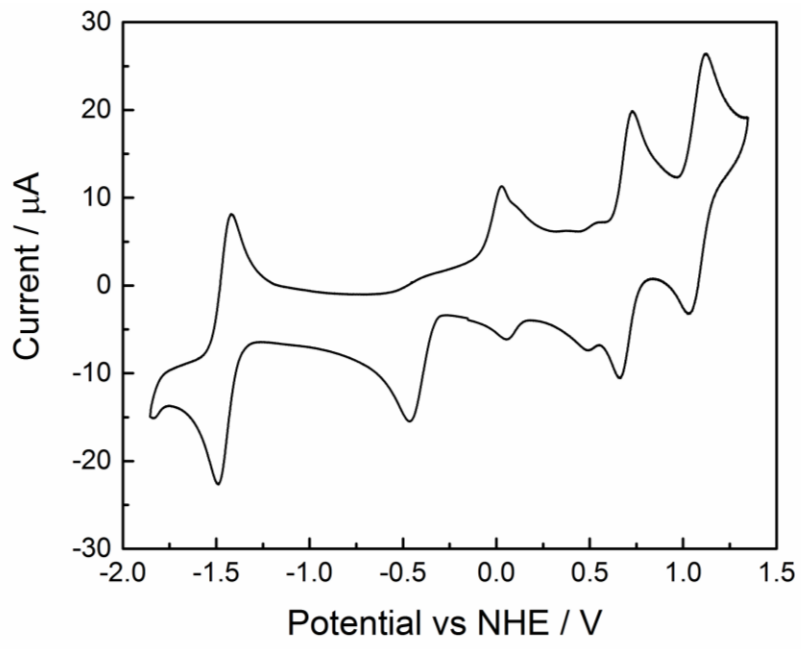

Supplementary Figure 7. Cyclic voltammetry of Co-Corrole in Ar dissolved in CH_3CN containing 0.1 M $TBAPF_6$ as supporting electrolyte with Ag/AgCl as reference electrode and glassy carbon as working electrode. Two metal centered redox peaks at -0.5 V (Co (III)/Co(II)) and -1.5 V (Co(II)/Co(I)) vs NHE could be identified. The irreversibility of the redox peak at -0.5 V is due to the partial or complete loss of the PPh_3 ligand.

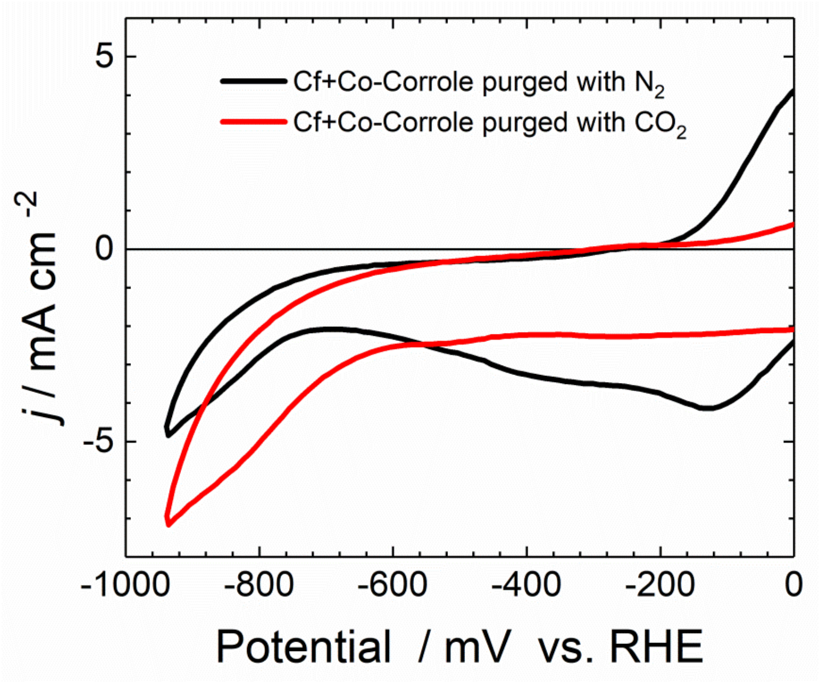

Supplementary Figure 8. Electrocatalytic CO_2 reduction: Cyclic voltammetry at pH = 6, phosphate buffer, 0.1 M $NaClO_4$.

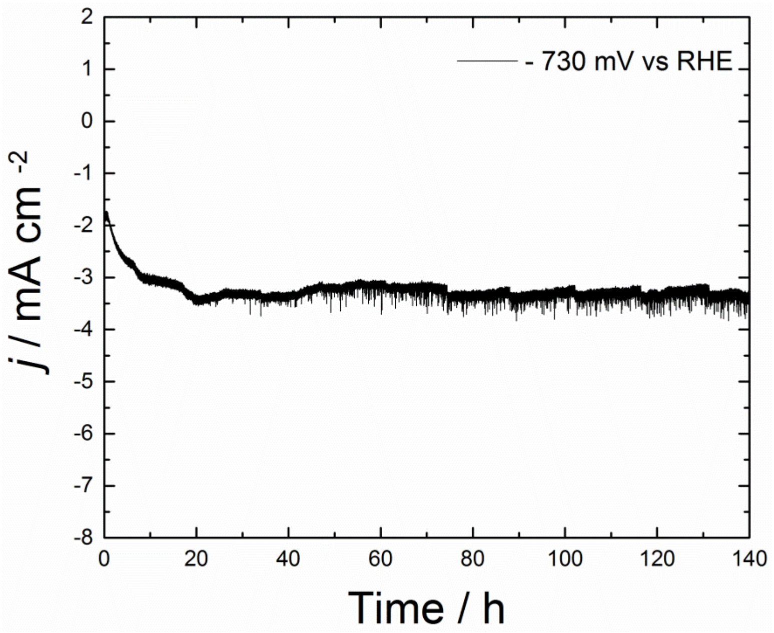

Supplementary Figure 9. Electrocatalytic CO_2 reduction at pH=6: Current density vs time during CO_2 dosage.

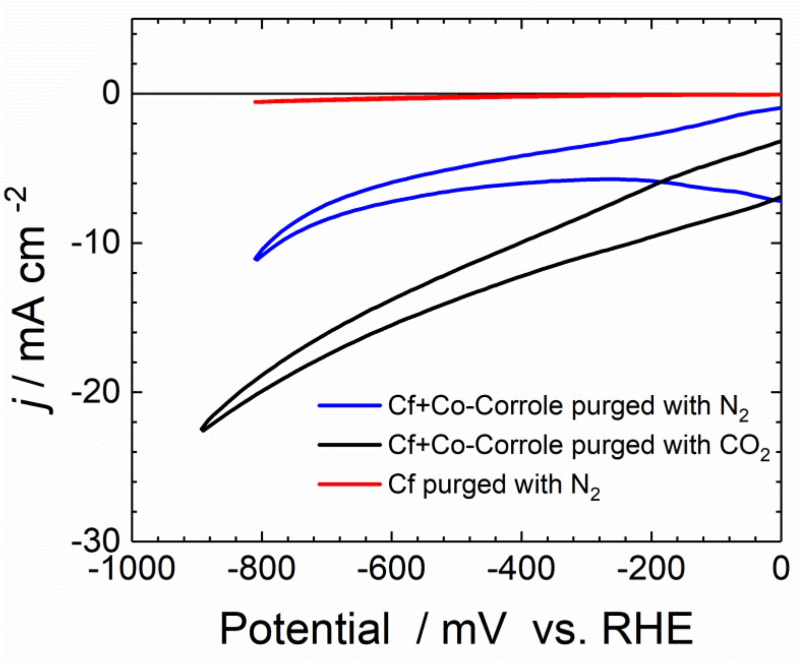

Supplementary Figure 10. Electrocatalytic CO_2 reduction: Cyclic voltammetry at pH = 7.2, 1 M $KHCO_3$

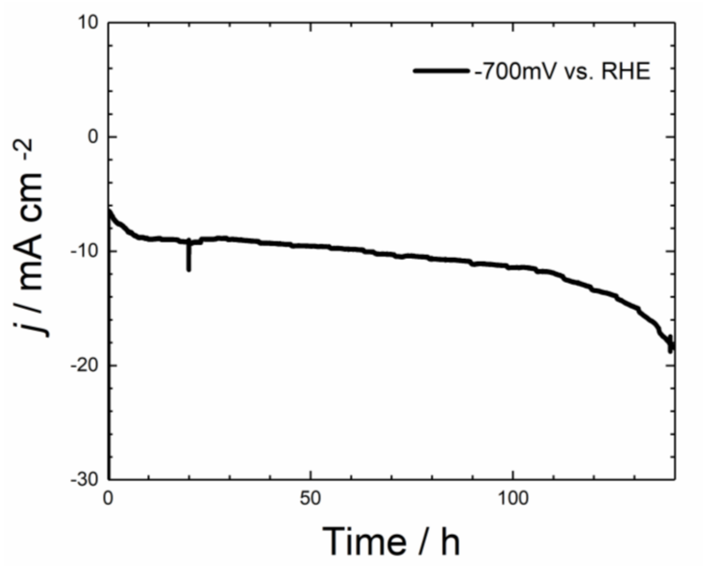

Supplementary Figure 11. Electrocatalytic CO_2 reduction at pH=7.2 and -0.7 V vs. RHE: Current density vs time during CO_2 dosage.

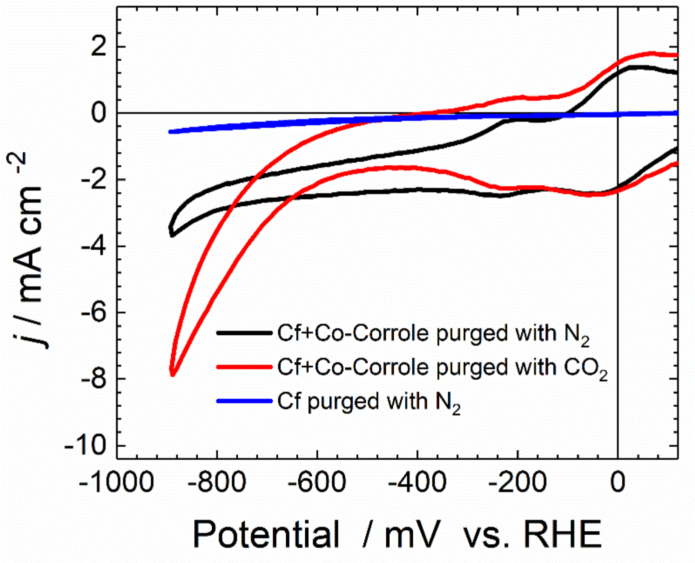

Supplementary Figure 12. Electrocatalytic CO_2 reduction: Cyclic voltammetry at pH = 8, 0.05 M $Na_2{}^{13}CO_3/NaH^{13}CO_3$

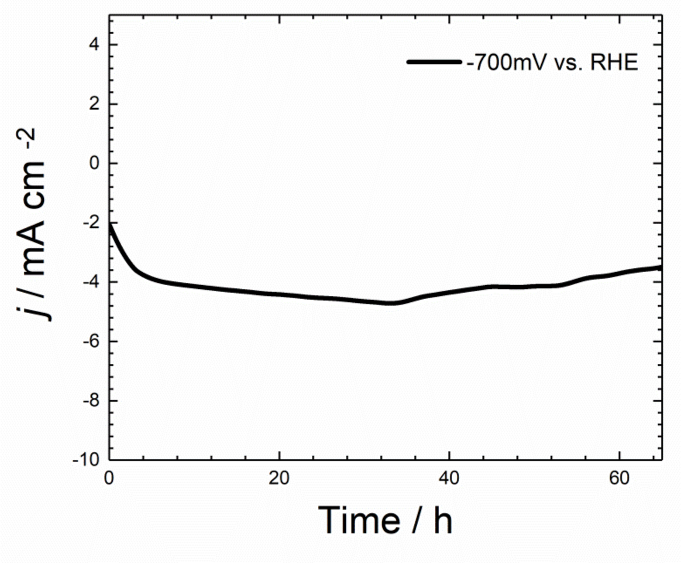

Supplementary Figure 13. Electrocatalytic CO_2 reduction at pH = 8: Current density vs time during CO_2 dosage.

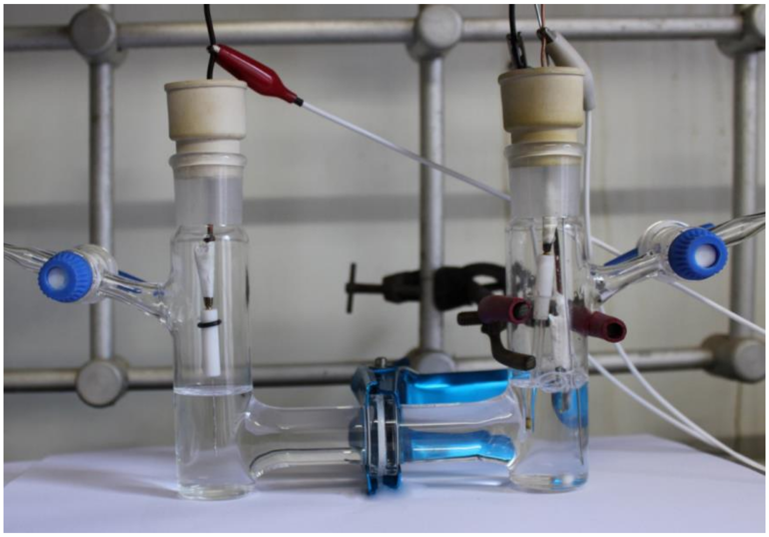

Supplementary Figure 14. Electrocatalytic CO_2 reduction: Two-compartment three- electrode electrochemical H-cell for CO_2 reduction reaction used in this work.

Supplementary Table 2. XPS data before the electrocatalysis reactions.

Name	Peak BE	Height CP:	FWHM eV	Area (P) C	Atomic %
C1s	285,05	29097,23	2,68	89822,6	79,82
O1s	532,26	12356,85	2,75	40785,68	13,41
F1s	687,85	4099,2	2,35	11453,17	2,67
Si2p	102,11	754,49	2,13	1769,53	1,84
S2p	164,23	713,89	2,49	2166,38	1,12
N1s	398,86	690,28	1,71	1558,45	0,8
Co2p	781,64	435,95	6,07	4269,27	0,24
P2p	132,31	134,73	0,19	156,11	0,11

Supplementary Table 3. XPS data after the electrocatalysis reactions.

Name	Peak BE	Height CP:	FWHM eV	Area (P) C	Atomic %
C1s	285,01	29122,92	2,53	86477,03	80,56
O1s	532,22	11813,9	2,71	36832,98	12,7
F1s	687,79	3248,12	2,36	8563,17	2,09
Si2p	102,1	980,29	1,63	1799,98	1,96
N1s	398,38	515,73	5,7	2720,29	1,46
S2p	164,12	514,86	2,54	1277,67	0,69
P2p	132,12	255,74	1,67	551,65	0,41
Co2p	781,41	469,91	1,73	2071,03	0,12

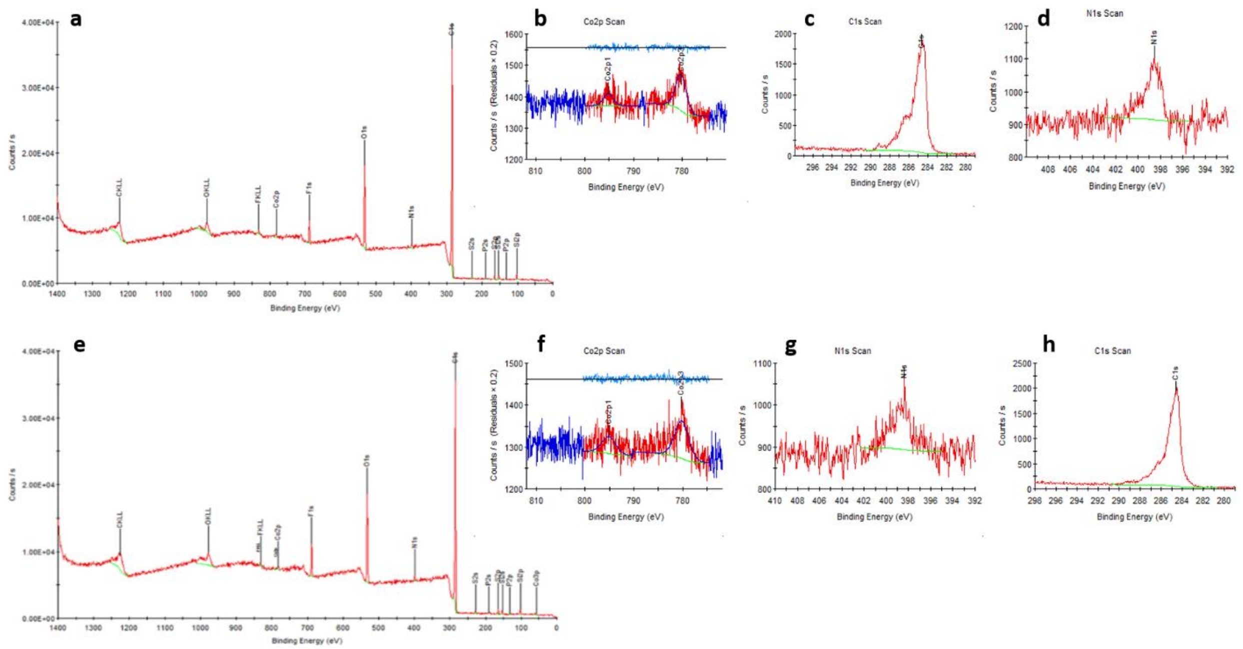

Supplementary Figure 15. a XPS survey scan spectra of the cobalt corrole on graphite before the electrocatalysis reactions. **b-d** High resolution XPS spectra of the Co-Corrole on carbon electrode before the electrocatalysis reaction corresponding **b** Co2p, **c** N1s, and **d** C1s binding energy regions and **e** XPS survey scan spectra of the cobalt corrole on graphite after the electrocatalysis reactions. **f-h** High resolution XPS spectra of the Co-Corrole on carbon electrode after the electrocatalysis reaction corresponding **f** Co2p, **g** N1s, and **h** C1s binding energy regions and the peak quantification of Co to N (ratio Co/N = ¼) indicating that the cobalt ion is located in the center of the corrole macrocycle and the Co-Corrole is still adsorbed on the electrode surface in an unmodified way.

Supplementary Note - 1: Determination of FE_{EtOH}(%)

For quantification of the products, a 5 point calibration curve was constructed with respect to the peak area obtained in the ^{1}H-NMR spectrum. More specifically, the triplet $-CH_3$ signal of ethanol at δ = 1.17 ppm was integrated to obtain a peak area vs concentration plot for standard ethanol solutions in the concentration range 0.1 mM to 10 mM (Supplementary Figure 16).

The slope of the calibration curve is 223.5 M^{-1}. Using the calibration curve, the concentration of ethanol in the electrolyte was determined to be 0.614 mM.

Total electrolyte volume is 30 mL. Therefore, the total amount of ethanol formed after 5 h of electrolysis at -0.800 V vs RHE is 1.84×10^{-5} moles.

Faradaic efficiencies (FE%) towards different products were calculated with the following equation;

$$FE\% = \frac{amount\ of\ product\ \times\ n\ \times\ F}{Q} \times 100$$

Where n = Number of electrons involved in formation of 1 product molecule

F = Faradaic constant

Q = Amount of charge passed through the working electrode (please refere to Supplementary Figure 19 → 44.48 C/18000s = 2.47 mA)

Thus, for ethanol,

$$FE\% = \frac{1.84\times 10^{-5}\ moles\times 12\times 96485.3\ \ s\,A\,mol^{-1}}{2.47\times 10^{-3}\ \ A\times 18000\ s} \times 100$$

Therefore, **FE% = 47.9 %**

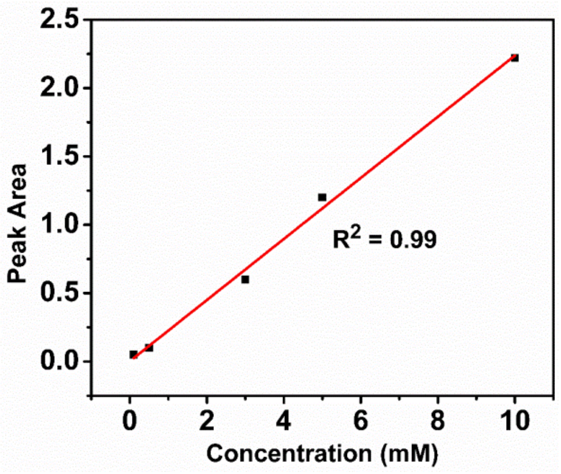

Supplementary Figure 16. Standard curve for ethanol constructed with authentic standards.

We integrated the ^{1}H-NMR resonance at 7.3 ppm (phenol, *m*-Hs, integral = 2Hs) and compared it to the signal at 3.65 ppm (EtOH, $-CH_2-$, *q*, integral = 2H,). We observed an integral ratio int. $_{phenol}$/int. $_{EtOH}$ = 4.7 (Supplementary Figure 17). We quantified all other reduction products accordingly (at different potentials, please see Supplementary Figure 18).

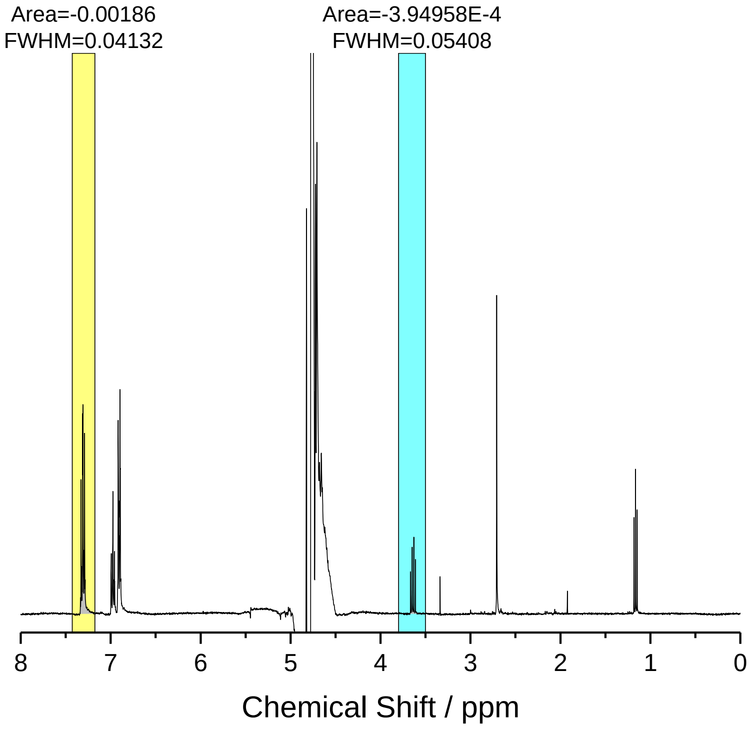

Supplementary Figure 17. ^{1}H-NMR spectrum of the liquid products formed during the heterogeneous reduction of CO_2 by Co-Corrole modified carbon paper electrode (0.1 M $NaClO_4$, pH=6, 0.1 M phosphate buffer hours, -0.8 V vs RHE. Quantification of ethanol: Integrals of ^{1}H-NMR resonances of the methylene protons of ethanol are referenced to phenol standard (signal at 7.3 ppm, 2Hs, ratio$_{\int phenol/\int ethanol}$ = 4.7).

20 mL of 20 mM phenol in DMSO
50 µL phenolic solution into NMR-tube = 0.0004 mol phenol/400 = $1 \cdot 10^{-6}$ mol phenol
Int. ratio phenol/EtOH = 4.7
$1x10^{-6}/4.7 = 2.13x10^{-7}$ mole EtOH.
$V_{electrolyte}$ = 30 mL → 350 µL in NMR-tube
30 mL/0.35 = 85.7

- → $2.13 \cdot 10^{-7}$ x 85.7= <u>$1.82x10^{-5}$ mol EtOH produced.</u> → **FE(%) = 47.4 %**

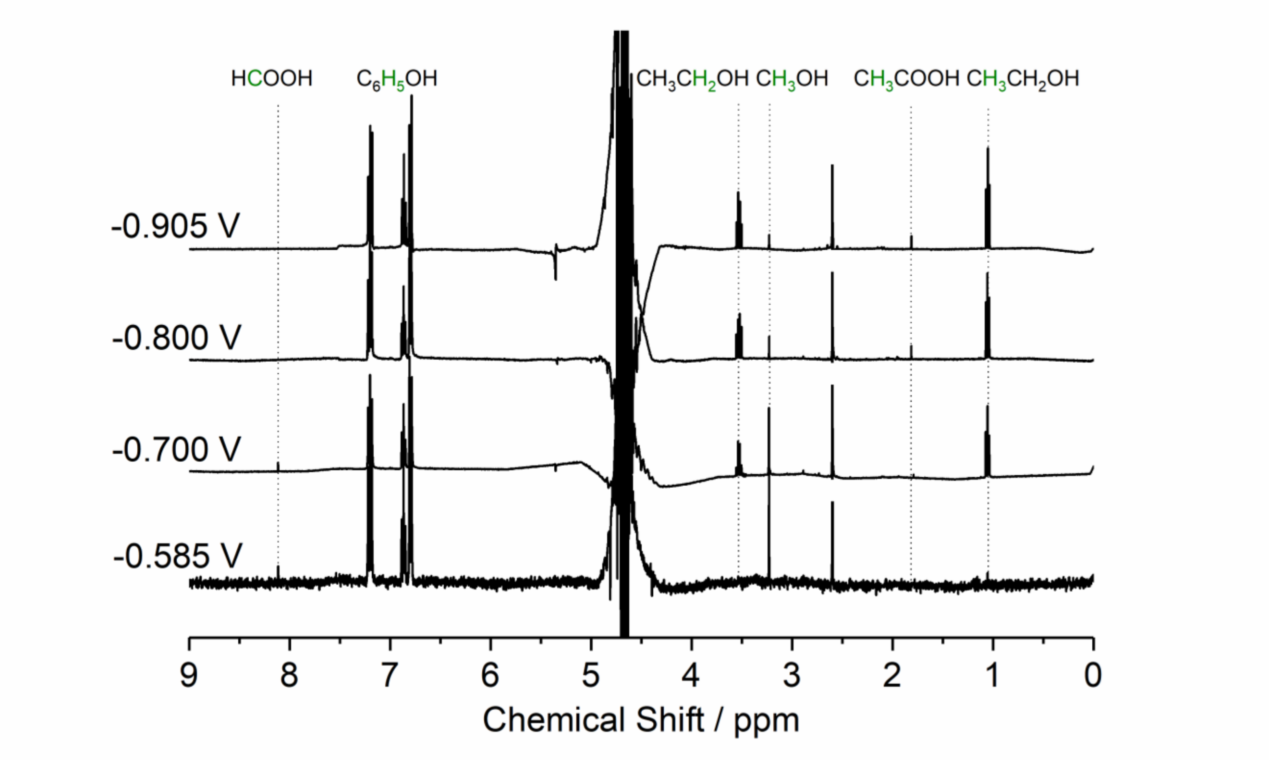

Supplementary Figure 18. Stacked plots of ^{1}H-NMR spectra observed after the electroreduction reaction at -0.585 V, -0.700 V, -0.800 V, and -0.905 V vs. RHE.

Supplementary Note - 2: Turn Over Frequency Calculations:

Turn Over Frequency (TOF) was calculated using the equation;[11]

$$\text{TOF} = \frac{iE_F}{NFn_{cat}}$$

$$\text{TOF} = 0.011\ s^{-1} \text{ or } 39.24\ hr^{-1}$$

Where i = current

E_F = Faradaic efficiency for ethanol

N = Number of electrons in the half reaction (N = 12 for CO_2 to ethanol conversion)

F = Faraday constant

n_{cat} = total moles of the catalyst employed for the electrolysis

Supplementary Note - 3: Turn Over Number Calculations:

Turn Over Number was calculated using the equation;

$$\text{TON} = \text{TOF} \times \text{t}$$

TON = 196.2

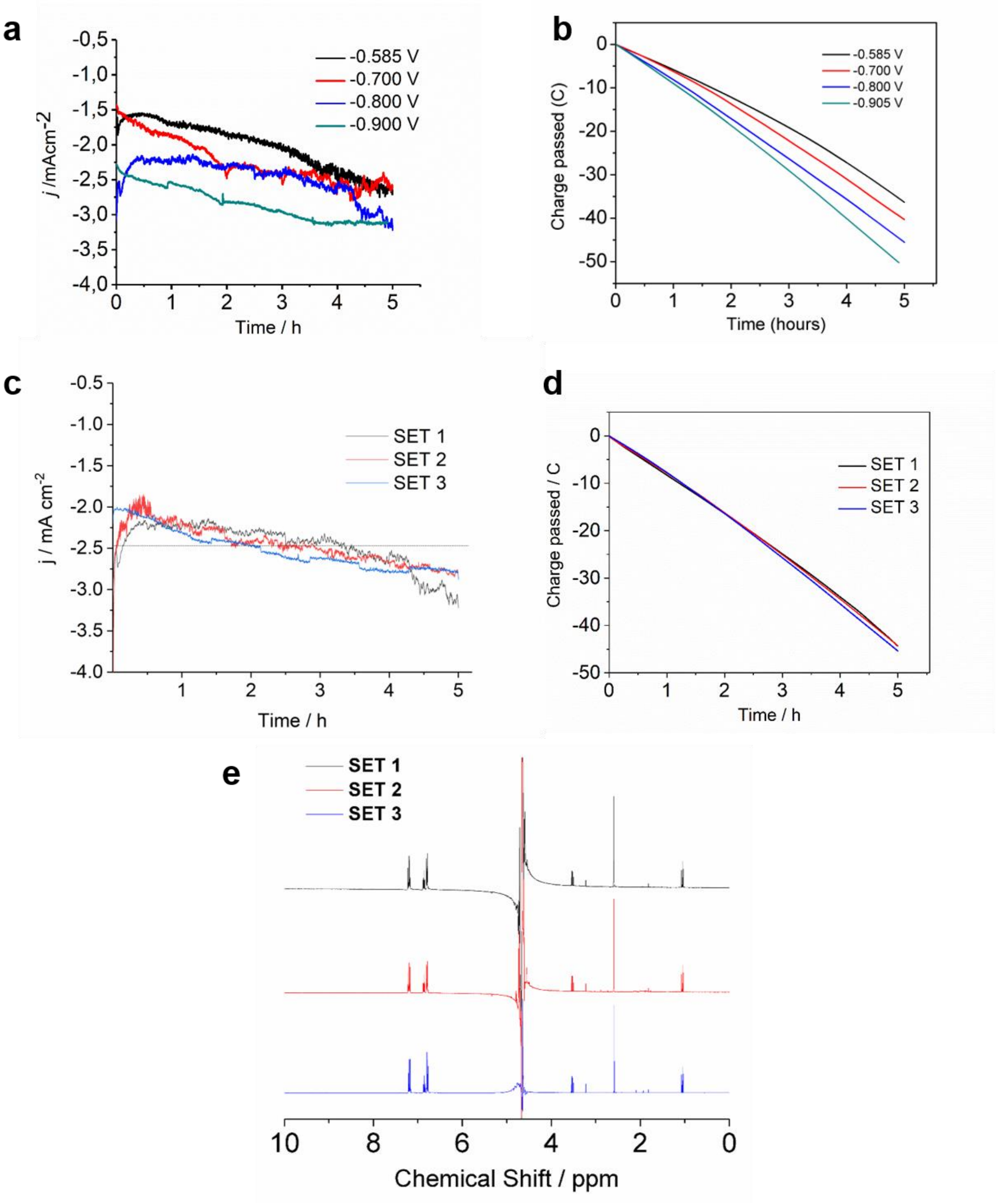

Supplementary Figure 19. a Controlled potential electrolysis data for the electrochemical CO_2 reduction by the Co-Corrole modified carbon paper electrode at four potentials -0.585 V vs RHE (black curve), -0.700 V vs RHE (red curve), -0.800 V vs RHE (blue curve) and -0.905 V vs RHE (green curve) and **b** charged passed vs time plot for the controlled potential electrolysis by the Co-Corrole modified carbon paper electrode at four potentials -0.585 V vs RHE (area = 36.27), -0.700 V vs RHE (area = 40.32), -0.800 V vs RHE (area = 44.48) and -0.905 V vs RHE (50.35). Data reproducability - **c** Three sets of controlled potential electrolysis data for the electrochemical CO_2 reduction by the Co-Corrole modified carbon paper electrode at -0.800 V vs RHE, **d** respective charged passed vs time plots for the controlled potential electrolysis by the Co-Corrole modified carbon paper electrode at -0.800 V vs RHE, and **e** the respective ^{1}H-NMR spectral analyses of all three sets 1-3.

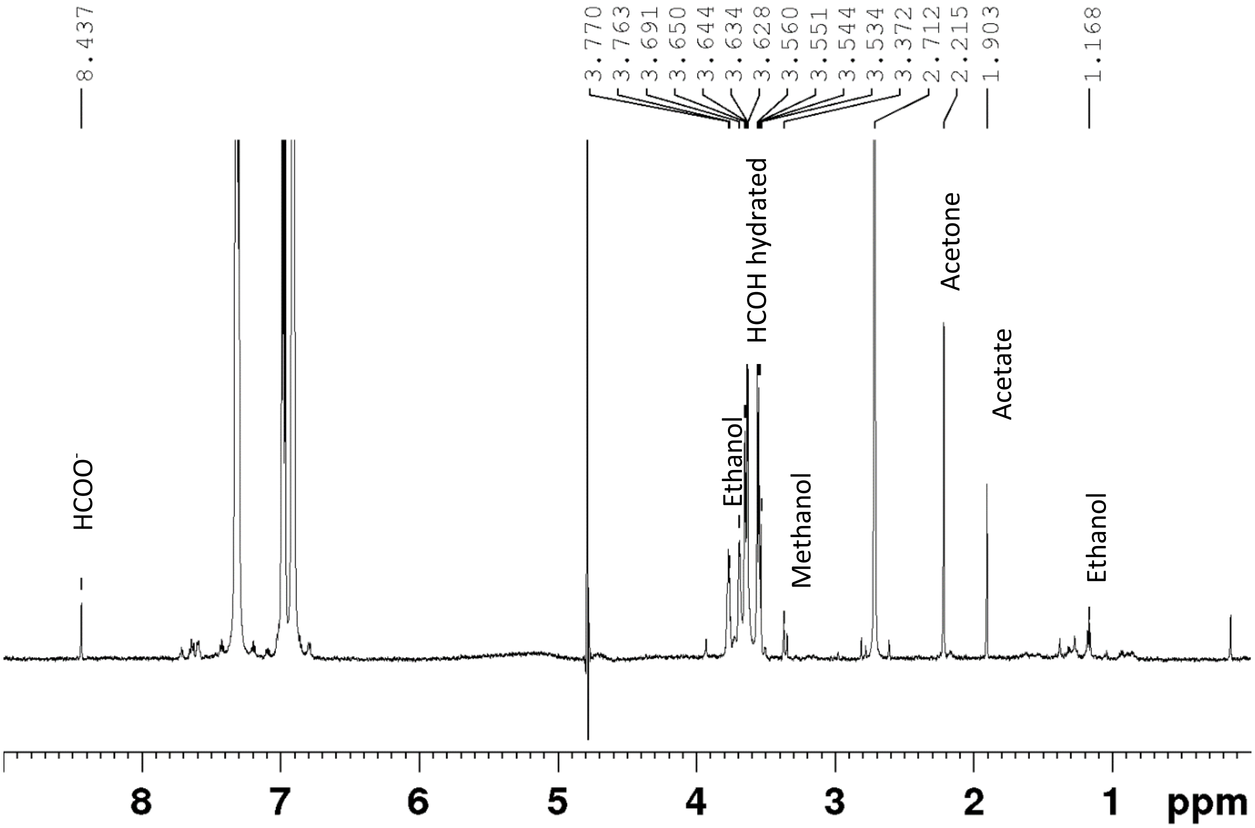

Supplementary Figure 20. ^{1}H-NMR spectrum of the liquid products formed during the heterogeneous CO_2 reduction by Co-Corrole modified carbon paper electrode (0.1 M $NaH^{13}CO_3$, pH = 7.2, 5 hours, -0.73 V vs RHE, phenol as internal standard)

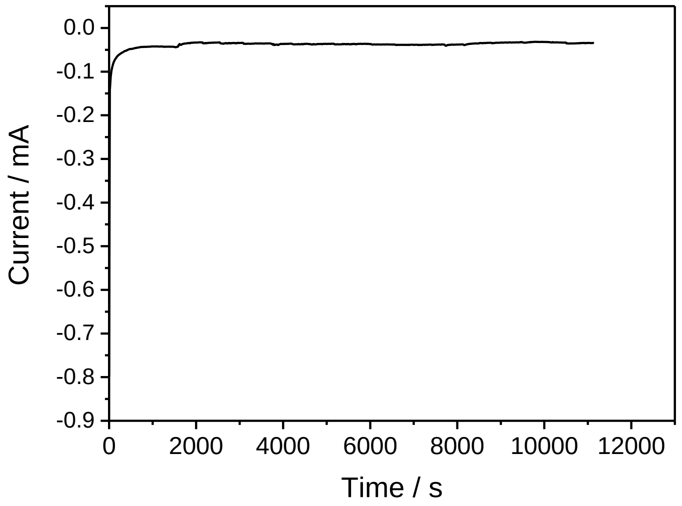

Supplementary Figure 21. i-t curve during electrocatalysis reaction under Argon (-0.8 V vs. RHE).

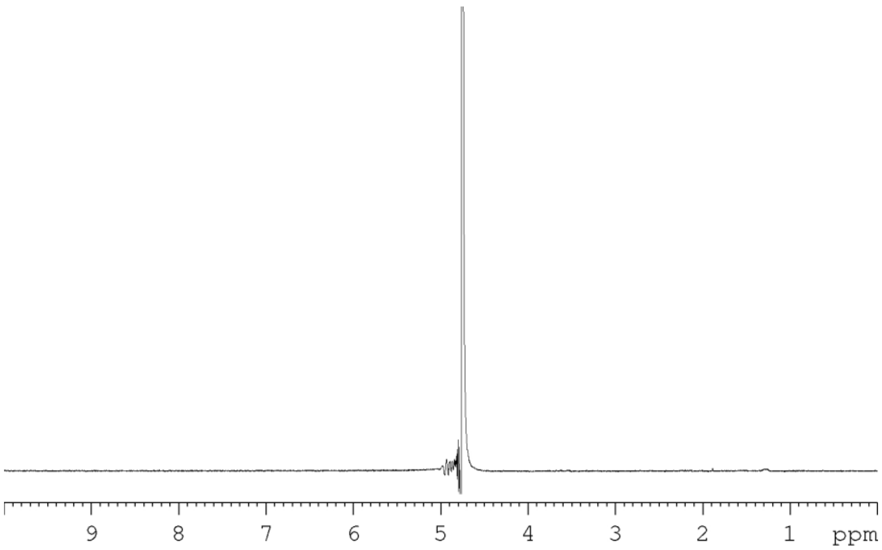

Supplementary Figure 22. ^{1}H-NMR spectrum after electrocatalysis reaction under Argon (540 µL electrolyte, 60 µL D_2O).

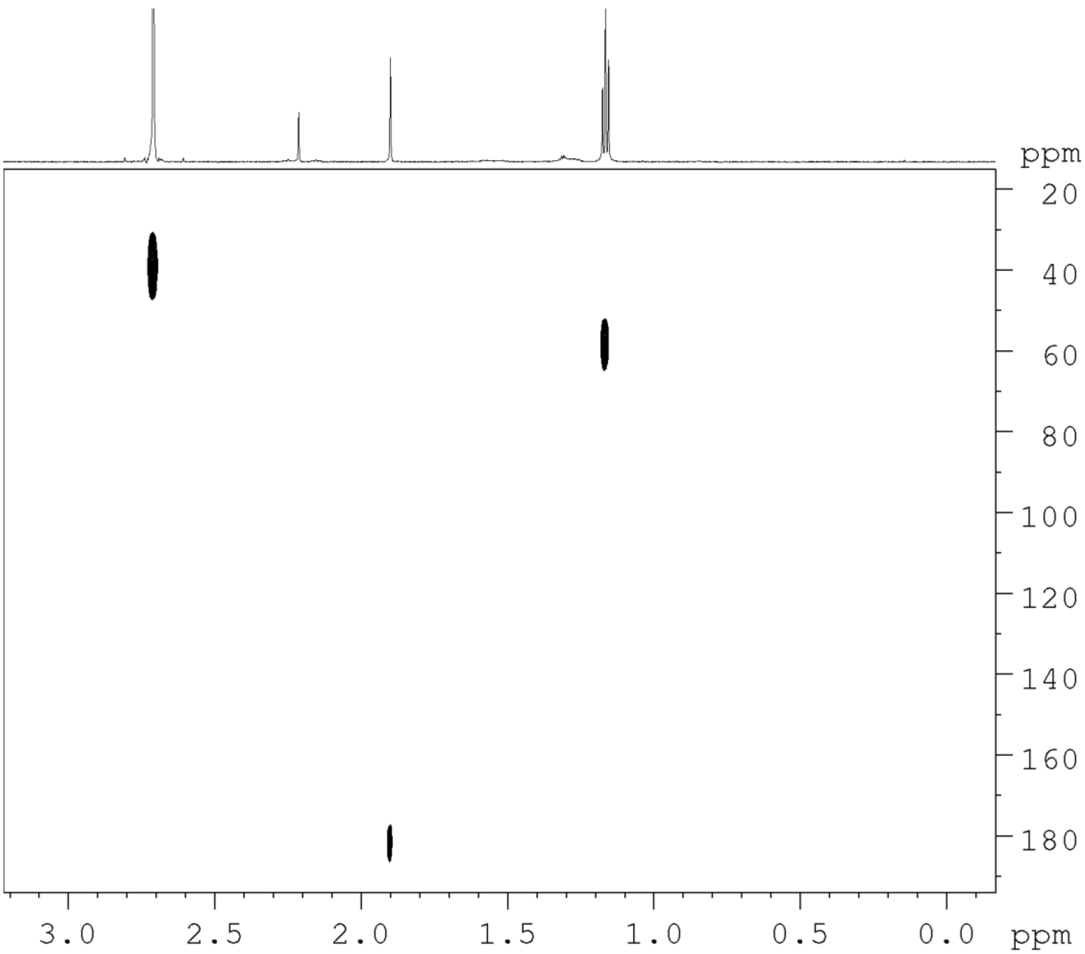

Supplementary Figure 23. Assignment of ^{1}H-NMR resonance at 1.9 ppm (acetate) *via* ^{1}H-^{13}C HMBC (one-bond C-H coupling constant and long-range coupling constant are adjusted to $^{1}J_{CH}$ = 130 Hz and $^{3}J_{CH}$ = 7 Hz, respectively).

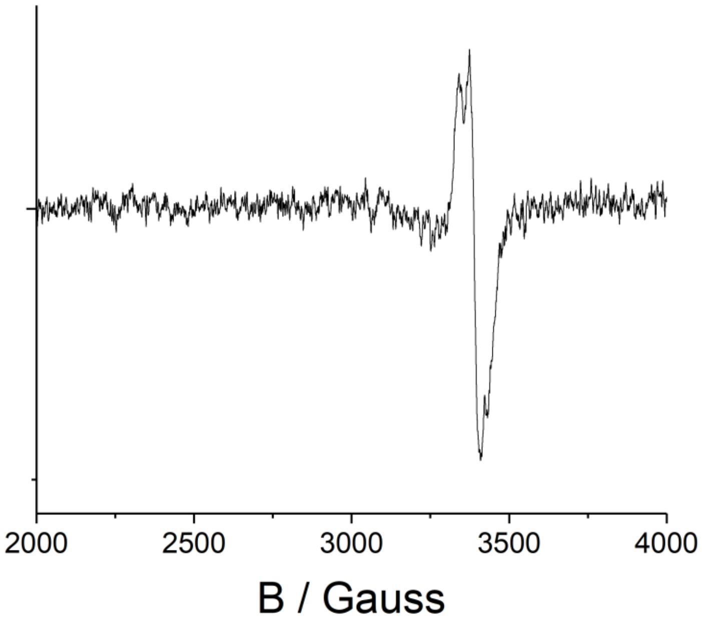

Supplementary Figure 24. EPR spectrum of Co-Corrole after electrochemical reduction and subsequent dosage of CO_2 indicating a rhombic S = 1/2 signal at g = 2 with a weak ^{59}Co hyperfine splitting.

Supplementary Note - 4: Control Experiments in CO_2 reduction

To confirm the involvement of radical intermediates like $CO_2H^•$, $CHO^•$, *etc;* experiments were repeated in the presence of radical quencher TEMPO (100 μM). No direct evidence of radical could be obtained from EPR spectroscopy but the addition of TEMPO quenched the reduction to CH_3CH_2OH confirming the formation of free radicals in the concerted CO_2 reduction pathway.

In a basic condition, the equilibrium existing between the two species (Co(III)-COOH and Co(III)-COO$^-$) shifts more towards Co(III)-COO$^-$ type intermediate. From this step the reaction can take two pathways, one is the CO pathway and the other is the formic acid pathway. It has already been established that a high concentration of bicarbonate ions favors the CO pathway.[12] In our case a decrease in CO_2 reduction towards ethanol in bicarbonate medium indicates the involvement of formic acid pathway over CO pathway.

To probe the reduction pathway for the formation of ethanol from CO_2, we have conducted CO_2 electrolysis in 0.1 M $NaHCO_3$ buffer (pH = 8). The cyclic voltammetry measurements show only an insignificant difference in current density profile for measurements done under both Ar and CO_2 environment (Supplementary Figure 25 A). Upon controlled potential electrolysis at -0.73 V vs RHE for 4000 s, absence of ethanol was observed. This negative dependence of CO_2 reduction on the hydroxyl anion concentration can also be inferred from equation S4. It is already reported in systems like Co-protoporphyrin that with increase in pH from 1 to 3 the CO production increases by many folds.[13] In our case a decrease in CO_2 reduction towards ethanol in bicarbonate medium indicates the involvement of formic acid pathway over CO pathway. Based on our experimental results, the CO formation is not favored in our catalysis (pH = 6), thus the CO_2 reduction proceeds *via* formic acid intermediate. But under basic condition (pH > 8) the formic acid pathway is disfavoured, where the CO_2 reduction to CO is observed (Supplementary Figure 25 B), which accounts for the absence of ethanol as depicted in ^{1}H-NMR data (Supplementary Figure 25 C).

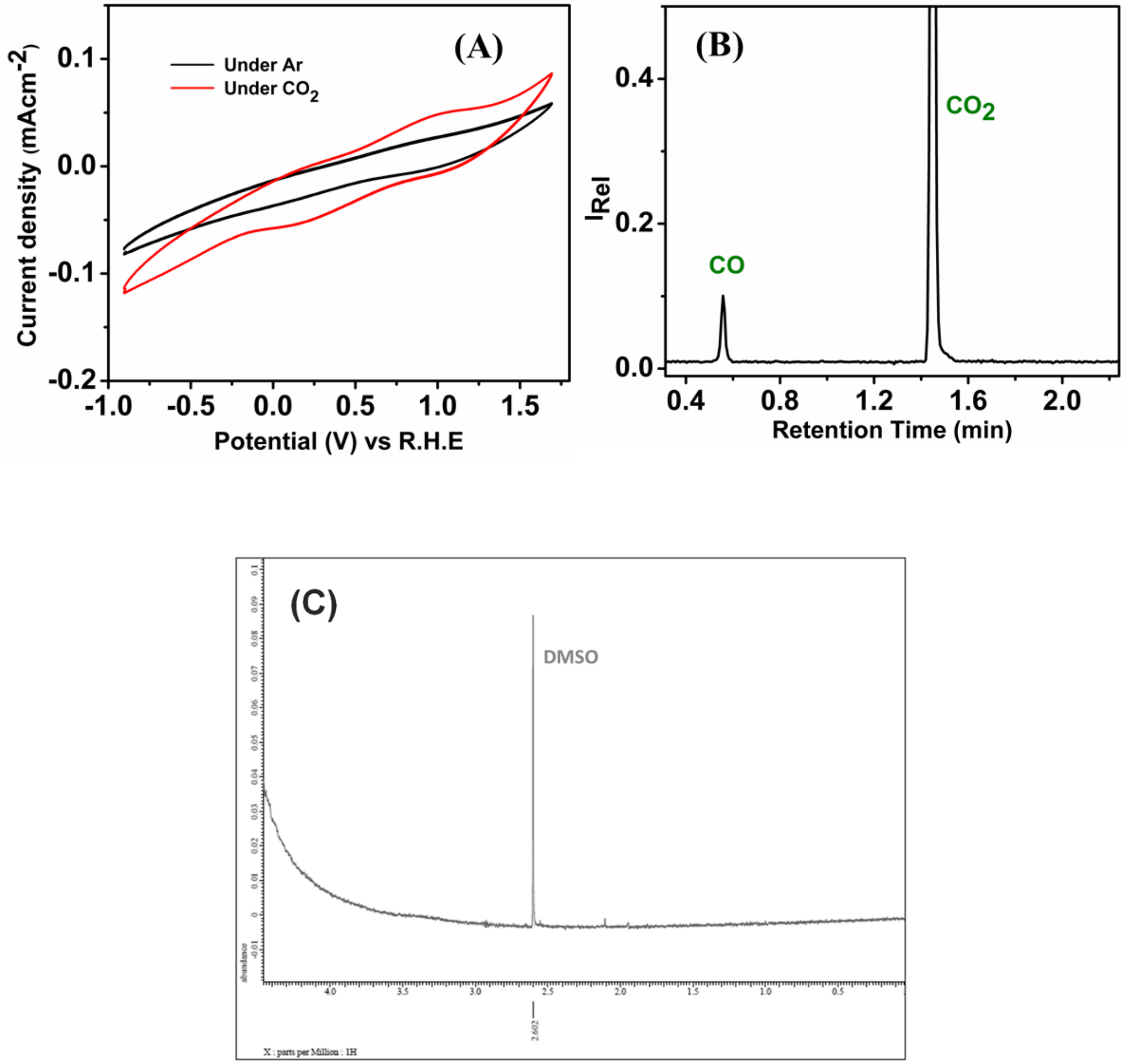

Supplementary Figure 25. (A) CV of the Co-Corrole-carbon paper electrodes at 100 mV s^{-1} in 0.1 M $NaClO_4$ (pH = 8.0, 0.1 M $NaHCO_3$ buffer) under Ar and CO_2 atmosphere. (B) Gaseous products obtained after 5 h of CO_2 electrolysis at -0.73 V vs RHE (pH = 8, 0.1 M $NaHCO_3$ buffer) with Co-corrole carbon paper electrodes showing presence of CO formation. (C) ^{1}H-NMR spectrum of the electrolyte after 5 h of controlled potential electrolysis of Co-Corrole-carbon paper (pH = 8, 0.1 M $NaHCO_3$ buffer) at -0.73 V vs RHE in CO_2 exhibits no methanol or ethanol formation.

Supplementary Note - 5: CO_2 electroreduction by Co-nanoparticles

Co-nanoparticles were synthesized by the addition of 225 µmol of $CoSO_4$ and 300 µmol of tetrabutyl ammonium bromide to 8 ml of water with stirring.[14] To this solution, 0.1 M $NaBH_4$ was added dropwise and the stirring was continued for 15 minutes and then the aqueous solution was decanted off. The precipitate of Co nanoparticles was further washed with water and acetone and dried under vacuum. 2 mg of this Co-nanoparticles where then sonicated for 10 minutes in 1 mL 10% aqueous nafion. Further, 500 µL of this dispersion was drop-casted over carbon paper electrode over 1 cm^2 area. On conducting similar experiments for CO_2 electroreduction (Supplementary Figure 26) no methanol or ethanol formation was observed as depicted in the ^{1}H-NMR spectrum in Supplementary Figure 27. Thus, from this control experiment it could be inferred that CO_2 reduction by Co-Corrole is completely a molecular property and not a property enforced by in-situ formation of Co metal nanoparticle.

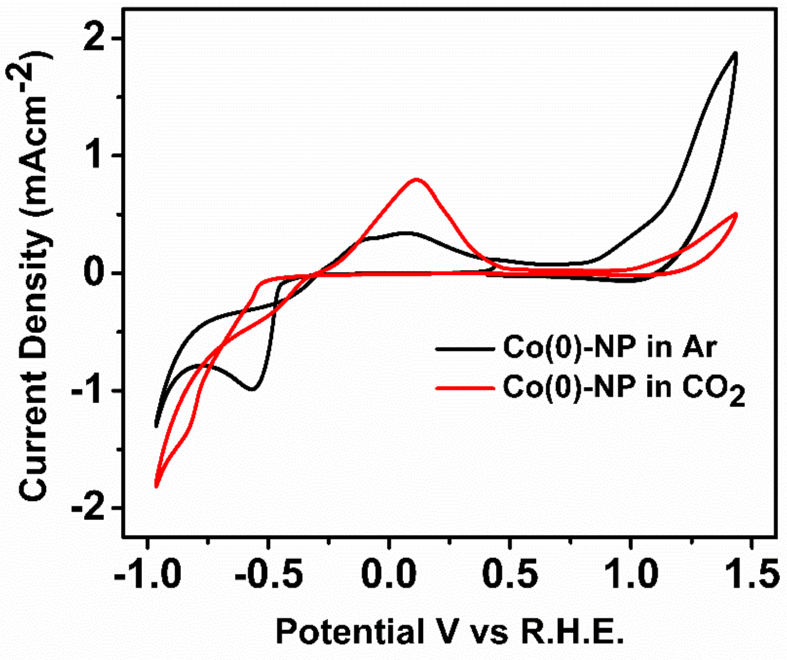

Supplementary Figure 26. CV of the Co-nanoparticles modified carbon paper electrodes at 100 mV s^{-1} in 0.1 M $NaClO_4$ (0.1 M pH = 6, phosphate buffer) under Ar and CO_2 atmosphere.

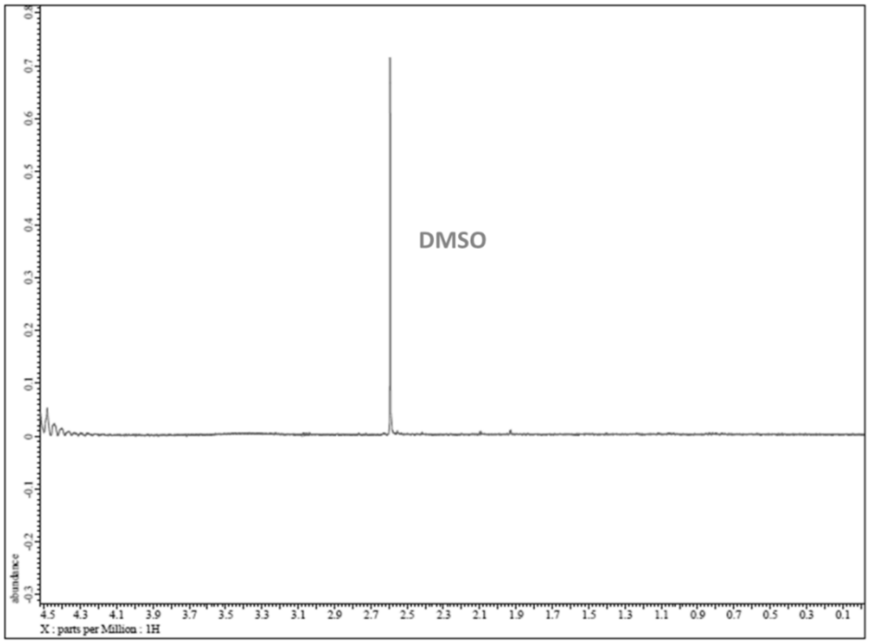

Supplementary Figure 27. ^{1}H-NMR spectrum of the reaction mixture after CO_2 electrolysis by Co-nanoparticles show the absence of formation of any methanol (at $\delta = 3.23$ ppm) or ethanol ($\delta = 1.05$ and $\delta = 3.5$ ppm respectively).

Supplementary Note - 6: DFT calculations

All the geometries were fully optimized by employing density functional theory (DFT) using a range separated hybrid functional wB97XD[15] which contains empirical dispersion correction term by Grimme[16] as implemented in Gaussian 09 quantum mechanical software package.[17] The geometry optimizations were carried out using a Pople basis set of valence triple ζ quality (6-311G)[18] over all the atoms (Co, S, P, O, N, C and H). We have optimized both low and high spin geometries for all the compounds. The solvent water was considered using the integral equation formalism of the polarized continuum model (IEF-PCM).[19] DFT calculations were done using a Co-Cor complex in which the PEG(7)-OMe unit was replaced with PEG(1)-OMe unit (Optimized Co(III)-Cor structure is given in Supplementary Figure 33 and the coordinates are given in ESI at page 44-49). Analytical vibrational frequencies were computed to verify the nature of the stationary states. The results obtained were used to calculate the binding energy differences between various geometries in the methanol and ethanol pathway. Only the Co-Corrole molecule was modeled and not the support electrode (carbon paper) since the latter doesn't influence the electroactivity. Further, theoretical redox potential calculations were carried out using self-consistent reaction field (SCRF) approach based on the integral equation formalism of the polarized continuum model (IEF-PCM) level of theory and the solvation free energies (ΔG_s^o) for the complex in all the oxidation states (Co(III), Co(II) and Co(I)) was found out using default options as given in Gaussian 09 (See page no: 41).[20]

On reduction of the metal center in Co-Corrole from Co(III) to Co(I), investigation of Mulliken charges of the two species involved in the reduction process (eqn. S1), revealed that charge on the metal center changed from +0.852 (Co(III)) to +0.799 (Co(I)) while the charges on nitrogen atoms of both the species have an average value of *ca*. -0.72.

In the next process, CO_2 molecule gets adsorbed onto the 2 e^- reduced Co(I) centre and the CO_2 molecule is having a linear arrangement (<O–C–O: 175.9°, C–O: 1.18 Å) which evidences the presence of a weak interaction (physisorption)[21] between CO_2 and the metal center (Supplementary Figure 28A) since the electronic structure of both moieties is not having appreciable perturbation. This initial adsorption is followed by the binding of CO_2 molecule *via* its C atom to the metal

center resulting in the formation of metal bound carboxyhydroxyl intermediate [Co(II)–COOH]$^{-}$ (Supplementary Note – 6, equation 3) where the protonated CO_2 molecule is found in a bent form (<O–C–O: 118.5°, C–O: 1.22 and 1.38 Å) (Supplementary Figure 28B).

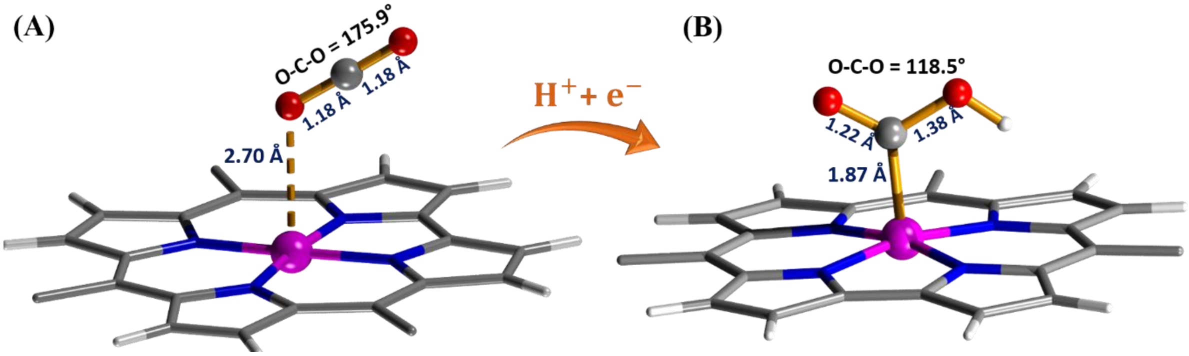

Supplementary Figure 28. Optimized structures of (A) [Co(I)-$CO_{2\ adsorbed}$]$^{2-}$ and (B) [Co(II)-COOH]$^{-}$ at wB91XD/6-311g level of theory (For clarity S-PEG(7)-OMe units are not shown).

The loss of linearity is due to the electron charge transfer from the metal center (Co(I)) to the CO_2 molecule which results in the orientation of the two CO bonds away from the metal containing plane with concomitant oxidation of Co(I) to Co(II). During this process, the charge on the metal center in [Co(II)–COOH]$^{-}$ changes to +0.824 which is intermediate to that of Co(III) and Co(I) and the charges on the carbon and oxygen atoms in CO_2 (C = +0.30, O = -0.41 and -0.58) were found to be greater than that of the free CO_2 (C = +1.05; O = -0.52 each). In order to understand the mechanism and origin of this charge transfer, the interaction between Co site of Co(II) Corrole and carboxyhydroxyl species on the [Co(II)-COOH]$^{-}$ intermediate was studied. On examination of HOMO orbital of [Co(II)-COOH]$^{-}$ (Supplementary Figure 29), a charge transfer transition between Co(II) d_{z^2} orbital (25.076%) to π* antibonding orbital (68.475%) of CO_2H was observed. A similar charge transfer was observed from Co(III) d_{z^2} orbital (34.591%) to π* antibonding orbital (45.464%) of CHO$^{\bullet}$ for Co(III)-CHO$^{\bullet}$ (Supplementary Figure 30).[22,23]

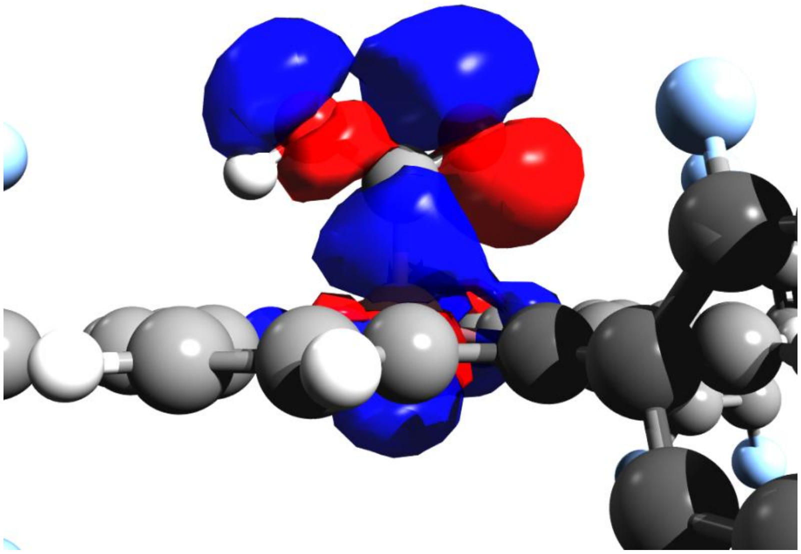

Supplementary Figure 29. Spatial representation of HOMO orbital of [Co(II)-COOH]$^{-}$ showing interaction of Co(II) with coordinated COOH$^{\bullet}$.

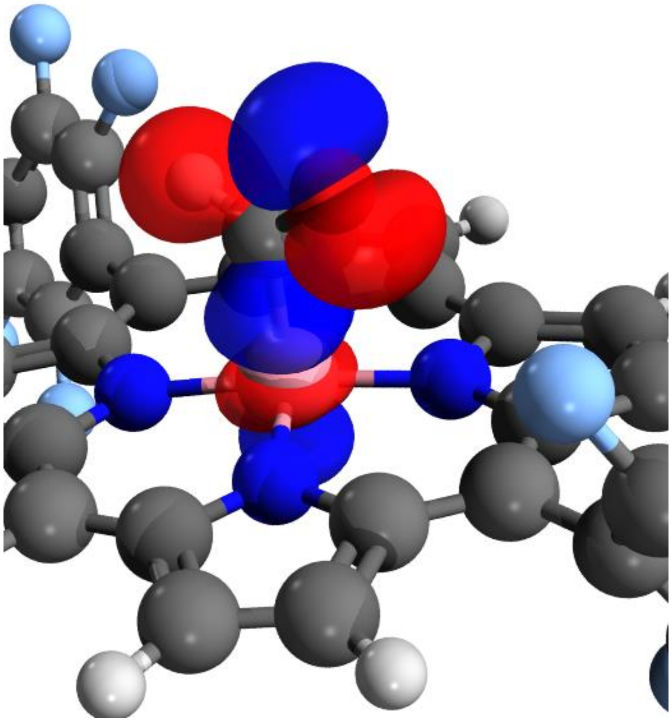

Supplementary Figure 30. Spatial representation of HOMO orbital of Co(III)-CHO$^{\bullet}$ showing interaction of Co(III) with coordinated CHO$^{\bullet}$.

Supplementary Note - 7: Possible equations involved in CO_2 electroreduction along with respective reaction free energies

$Co(III)PPh_3 + 2\,e^- \rightarrow [Co(I)]^{2-} + PPh_3$ ⋯⋯ (1) $\Delta G = -0.48\ eV$

$[Co(I)]^{2-} + H_2O \rightarrow [Co(I) - (OH_2)]^{2-}$ ⋯⋯ (2) $\Delta G = -0.12\ eV$

$[alternative\ (Hydride\ formation)\ \ Co(I)]^{2-} + H_2O \rightarrow [Co(III) - H]^- + OH^-$ ⋯⋯ (2.1) $\Delta G = +3.08\ eV$

$[Co(I)]^{2-} + CO_2 \rightarrow [Co(I) - CO_{2\,ads}]^{2-}$ ⋯⋯ (3) $\Delta G = +0.51\ eV$

$[Co(I) - CO_{2\,ads}]^{2-} + H_2O \rightarrow [Co(II) - COOH]^- + OH^-$ ⋯⋯ (4) $\Delta G = -1.00\ eV$

$[Co(II) - COOH]^- + H_2O \rightarrow [Co(III) - HCOOH]^0 + OH^-$ ⋯⋯ (5) $\Delta G = +0.13\ eV$

$[Energetically\ disfavored\ Co(III) - COOH]^0 + e^- \rightarrow [Co(III) - CO]^0 + OH^-$ ⋯⋯ (5.1) $\Delta G = +1.84\ eV$

$[Co(III) - HCOOH]^0 + e^- \rightarrow [Co(III) - HCO^{\cdot}]^0 + OH^-$ ⋯⋯ (6) $\Delta G = +0.45\ eV$

Methanol pathway

$Co(III) - HCO^{\cdot} + e^- + H_2O \rightarrow Co(III) - HCHO + OH^-$ ⋯⋯ (7) $\Delta G = -0.31\ eV$

$Co(III) - HCHO + e^- + H_2O \rightarrow Co(III) - OCH_3 + OH^-$ ⋯⋯ (8) $\Delta G = -1.93\ eV$

$Co(III) - OCH_3 + e^- + H_2O \rightarrow Co(III) + CH_3OH + OH^-$ ⋯⋯ (9) $\Delta G = +1.92\ eV$

Ethanol pathway

$Co(III) - HCO^{\cdot} + HCO^{\cdot} \rightarrow Co(III) - CHO - CHO$ ⋯⋯ (10) $\Delta G = -3.31\ eV$

$Co(III) - CHO - CHO + e^- + H_2O \rightarrow Co(III) - OCH_2 - CHO + OH^-$ ⋯⋯ (11) $\Delta G = +1.85\ eV$

$Co(III) - OCH_2 - CHO + 2e^- + 2H_2O \rightarrow Co(III) - OCH_2 - CH_2OH + 2OH^-$ ⋯⋯ (12) $\Delta G = -5.65\ eV$

$Co(III) - OCH_2 - CH_2OH \rightarrow Co(III) - OCH = CH_2 + H_2O$ ⋯⋯ (13) $\Delta G = +0.05\ eV$

$Co(III) - OCH = CH_2 + 2e^- + 2H_2O \rightarrow Co(III) - OCH_2 - CH_3 + 2OH^-$ ⋯⋯ (14) $\Delta G = +3.38\ eV$

$Co(III) - OCH_2 - CH_3 + e^- + H_2O \rightarrow Co(III) + HOCH_2 - CH_3 + OH^-$ ⋯⋯ (15) $\Delta G = -2.62\ eV$

ΔG = reaction free energy (298.15 K) computed at wB97XD/6-311G level of theory.

Supplementary Table 4. List of all the species involved in the catalytic process with their total electronic energies for low and high spin states along and the energy difference.

Species	Low spin / eV (Spin multiplicity)	High spin / eV (Spin multiplicity)	Energy difference / eV
$[L\text{-}Co(III)PPh_3]^0$	-175439.7739 (1)	-175438.5626 (3)	1.2113
$[L\text{-}Co(I)]^{2-}$	-147242.2392 (1)	-147243.2635 (3)	-1.0243
$[L\text{-}Co(III)\text{-}H]^-$	-147260.4339(1)	-147259.7163 (3)	0.7176
$[L\text{-}Co(I)\text{-}CO_{2\ adsorbed}]^{2-}$	-152372.6026 (1)	-152373.1453 (3)	-0.5427
$[L\text{-}Co(II)\text{-}COOH]^-$	-152390.7973 (1)	-152389.6368 (3)	1.1605
$[L\text{-}Co(III)\text{-}HCO_2H]^0$	-152403.8227 (1)	-152404.3295 (3)	-0.5067
$[L\text{-}Co(III)\text{-}CO]^0$	-150324.0097 (1)	-150323.1521 (3)	0.8577
$[L\text{-}Co(III)\text{-}CHO]^0$	-150339.7477 (2)	-150338.1594 (4)	1.5883
$[L\text{-}Co(III)\text{-}OCH_2]^0$	-150356.2048 (1)	-150356.6641 (3)	-0.4593
$[L\text{-}Co(III)\text{-}OCH_3]^0$	-150372.2960 (2)	-150372.2612 (4)	0.0348
$[L\text{-}Co(III)\text{-}CHO\text{-}CHO]^0$	-153439.2811 (1)	-153439.9005 (3)	-0.6194
$[L\text{-}Co(III)\text{-}OCH_2\text{-}CHO]^0$	-153455.6284 (2)	-153455.6233 (4)	0.0051
$[L\text{-}Co(III)\text{-}OCH_2\text{-}CH_2OH]^0$	-153488.6718 (2)	-153488.6142 (4)	0.0575
$[L\text{-}Co(III)\text{-}OCH{=}CH_2]^0$	-151408.9191 (2)	-151409.1769 (4)	-0.2578
$[L\text{-}Co(III)\text{-}OCH_2\text{-}CH_3]^0$	-151442.1527 (2)	-151442.1869 (4)	-0.0342

Supplementary Note - 8: Computational Redox Potential

The half-cell reaction corresponding to 1 electron reduction can be given as

Ox^{n} (solv) + e^{-} → Red^{n-1} (solv)

where Ox = oxidized species and Red = reduced species.

Now the corresponding thermodynamic cycle for Gibbs free energy calculation is given as follows

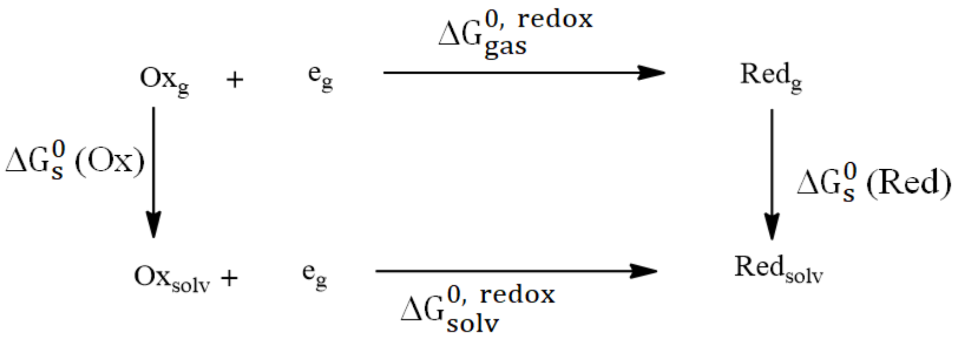

Supplementary Figure 31. Thermodynamic cycle for the calculation of Gibbs free energy for one electron reduction process.

$\Delta G_{solv}^{0,redox}$ which is the standard Gibbs free energy of redox half reaction in a solution can be calculated *via* the following equation

$$\Delta G_{solv}^{0,redox} = \Delta G_{gas}^{0,redox} + \Delta G_{s}^{0}(Red) - \Delta G_{s}^{0}(Ox)$$

Where $\Delta G_{gas}^{0,redox}$ is the free energy change in the gas phase, $\Delta G_{s}^{0}(Red)$ and $\Delta G_{s}^{0}(Ox)$ are the solvation free energies of the reduced and oxidized forms respectively.

Now $\Delta G_{solv}^{0,redox} = -F \times E_{calc}^{0}$;

where F = 23.06 kcal $mol^{-1}V^{-1}$ and E_{calc}^{0} = standard one electron reduction potential.

$$E_{calc}^{0} \text{ vs (SHE)} = E_{calc}^{0} - E_{SHE}^{0}$$

Where E^0_{SHE} = absolute potential of the standard hydrogen electrode, in acetonitrile solvent it is reported to be at 4.429 V[24] and $E_{exp,Ag/AgCl}$= experimental redox potential of Ag/AgCl couple which is 0.291 V relative to the standard hydrogen electrode.

E_{calc} (V vs RHE) = $E^0_{calc} - E^0_{SHE}$ + 0.0592 V × pH

Absolute potential for standard hydrogen electrode in water, E^0_{SHE} = -4.281 V

For one electron reduction of Co-corrole^0 → Co-corrole^{1-}

$E^{0,\ Co(III)\rightarrow Co(II)}_{calc}$= +3.971 V

$E^{Co(III)\rightarrow Co(II)}_{calc}$ vs (NHE) = -0.458 V (Experimental value: -0.50 V)

$E^{Co(III)\rightarrow Co(II)}_{calc}$ vs (RHE) = -0.01 V (Experimental value: -0.1 V)

For one electron reduction of Co-corrole^{1-} → Co-corrole^{2-}

$E^{0,\ Co(II)\rightarrow Co(I)}_{calc}$= +3.050 V

$E^{Co(II)\rightarrow Co(I)}_{calc}$ vs (NHE) = -1.379 V (Experimental value -1.50 V)

E_{calc} (V vs RHE) = $E^0_{calc} - E^0_{SHE}$ + 0.0592 V × pH = 3.05 – 4.28 + 0.0592*6 = -0.877 V (Experimental value: -0.8 V)

Supplementary Table 5. Theoretical bond lengths around the Co central core in [Co-Corrole]^{0}, [Co-Corrole]$^{1-}$, [Co-Corrole]$^{2-}$ in air and water.

	Co-N1 (Å)	Co-N2 (Å)	Co-N3 (Å)	Co-N4 (Å)	Co-P (Å)
**[Co-Corrole]^{0} (air)**	1.878	1.887	1.903	1.906	2.256
**[Co-Corrole]^{0} (water)**	1.887	1.878	1.907	1.906	2.264
**[Co-Corrole]$^{1-}$ (air)**	1.855	1.855	1.892	1.892	4.337
**[Co-Corrole]$^{1-}$ (water)**	1.859	1.860	1.886	1.893	4.703
**[Co-Corrole]$^{2-}$ (air)**	1.859	1.860	1.886	1.893	4.470
**[Co-Corrole]$^{2-}$ (water)**	1.857	1.857	1.868	1.868	6.6225

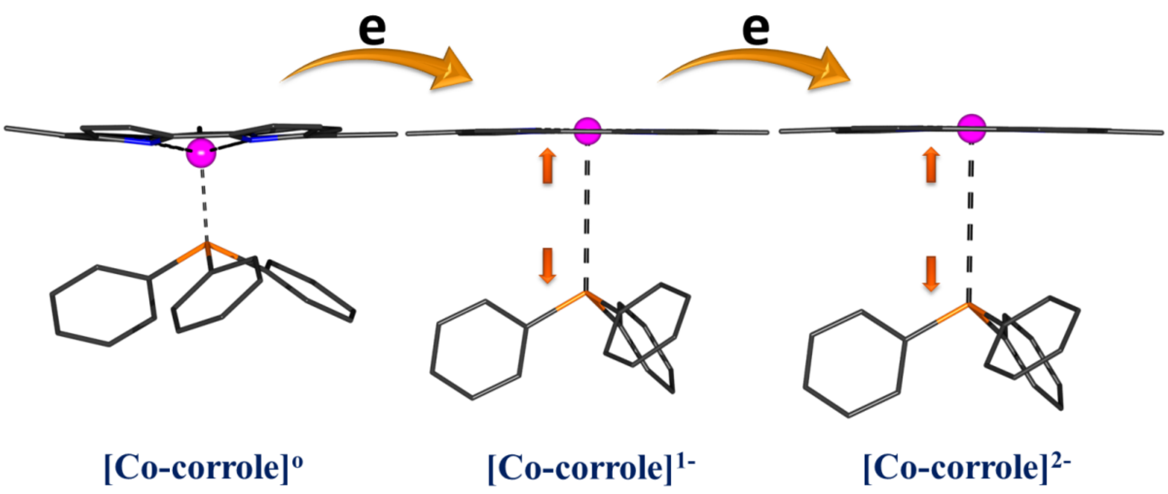

Supplementary Figure 32. DFT optimized geometries of [Co-corrole]^{0}, 1 e^- and 2 e^- reduced species showing the movement of Co center into the central cavity of the corrole ring with concomitant lengthening of Co-PPh_3 bond upon successive reduction.

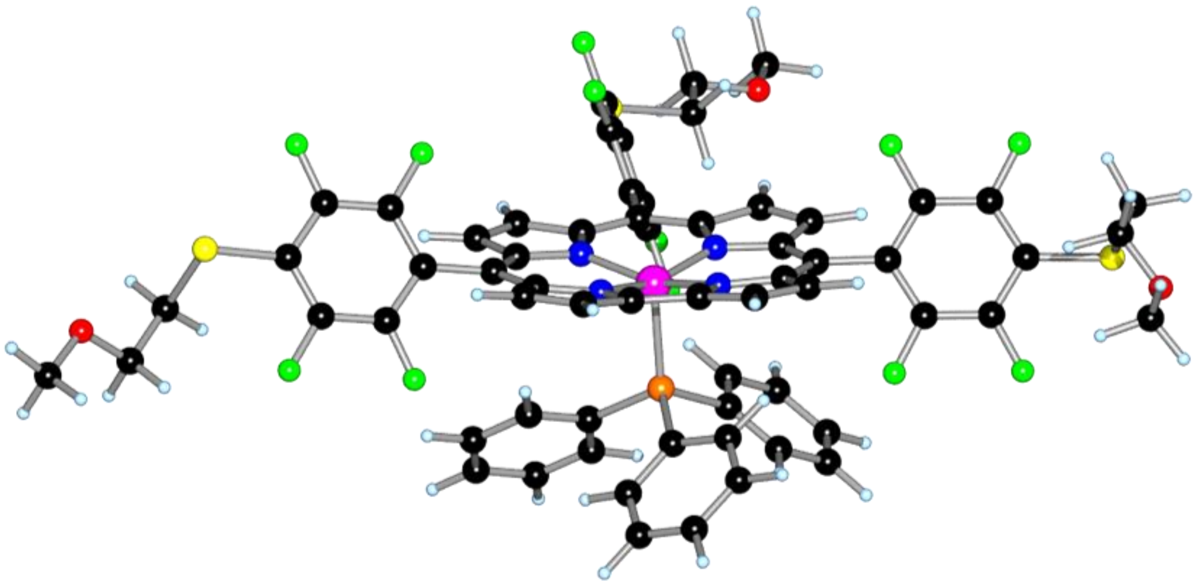

Supplementary Figure 33. Optimized structure of Co(III)-Cor at wB97XD/6-311g level of theory (pink = cobalt, blue = nitrogen, green = fluorine, orange = phosphorous, red = oxygen, black = carbon, pale blue = hydrogen).

Supplementary Table 6. Coordinates of optimized geometry of Co(III)-Corrole

Co	0.11308	-0.96712	-0.37574
N	-1.04265	-2.37416	-0.83565
N	-1.33584	0.25551	-0.54804
N	1.45185	0.37847	-0.55634
N	1.39887	-2.27470	-0.81968
C	-0.47801	-3.58282	-1.13941
C	-1.51557	-4.51354	-1.44070
C	-2.71466	-3.83650	-1.30497
C	-2.40610	-2.48354	-0.93078
C	-3.22141	-1.35309	-0.77182
C	-2.69008	-0.05652	-0.61740

C	-3.44674	1.16584	-0.59785
C	-2.56042	2.20346	-0.53833
C	-1.24170	1.63456	-0.49404
C	-0.03029	2.34050	-0.41425
C	1.23942	1.74243	-0.44177
C	2.50453	2.42362	-0.44750
C	3.47416	1.47283	-0.59554
C	2.82523	0.19287	-0.67910
C	3.46655	-1.04099	-0.91571
C	2.75895	-2.24900	-1.00660
C	3.17543	-3.56502	-1.40762
C	2.04273	-4.35400	-1.48504
C	0.93727	-3.52483	-1.13192
C	-4.69732	-1.54526	-0.79877
C	4.93830	-1.06580	-1.14038
C	-0.09816	3.83194	-0.36602
C	0.23501	4.61127	-1.47669
C	0.16275	6.00110	-1.45402
C	-0.24572	6.69007	-0.30761
C	-0.56826	5.91475	0.81125
C	-0.50638	4.52650	0.77549
F	0.62515	4.01650	-2.61702
F	0.48911	6.67003	-2.56982
S	-0.27083	8.46908	-0.27778
F	-0.94711	6.50666	1.95631

C	-5.50448	-1.06983	-1.83675
C	-6.88179	-1.25654	-1.83949
C	-7.53029	-1.96467	-0.82099
C	-6.72433	-2.45298	0.21294
C	-5.34838	-2.24397	0.22196
F	-4.95188	-0.39771	-2.86197
F	-7.58978	-0.74846	-2.86328
S	-9.29878	-2.16745	-0.76812
F	-7.26638	-3.13322	1.23460
F	-4.64192	-2.73371	1.25742
C	5.52749	-0.46149	-2.25542
C	6.90113	-0.49328	-2.47539
C	7.76823	-1.15000	-1.59601
C	7.18066	-1.77661	-0.49201
C	5.81045	-1.72547	-0.26862
F	4.76026	0.17927	-3.15424
F	7.38151	0.11904	-3.56894
S	9.50888	-1.24397	-1.94947
F	7.94691	-2.43787	0.39412
F	5.32831	-2.33537	0.83344
H	4.18999	-3.86018	-1.63998
H	1.98829	-5.39115	-1.78955
H	-1.37805	-5.54481	-1.73878
H	-3.70715	-4.22989	-1.48064
H	4.54203	1.63384	-0.65094

H	2.64216	3.49272	-0.36367
H	-4.52460	1.23255	-0.64629
H	-2.78717	3.26055	-0.53868
F	-0.83216	3.85168	1.89566
C	-9.62676	-3.09399	-2.33361
C	-9.15909	-4.53972	-2.33001
H	-9.18263	-2.54760	-3.16750
H	-10.71535	-3.05267	-2.43776
O	-9.89170	-5.26630	-1.36346
H	-8.07746	-4.59865	-2.12778
H	-9.32647	-4.95885	-3.33716
C	-9.48678	-6.62218	-1.28153
H	-9.63708	-7.14821	-2.23660
H	-10.10234	-7.09601	-0.51283
H	-8.42686	-6.71072	-0.99870
C	10.27038	-0.59310	-0.38328
C	11.15633	-1.62442	0.29032
H	9.47133	-0.27063	0.28546
H	10.86012	0.28241	-0.66661
O	12.21147	-1.94944	-0.59760
H	10.57378	-2.52016	0.55052
H	11.55601	-1.19607	1.22533
C	13.05763	-2.96788	-0.09061
H	13.53788	-2.66114	0.85079
H	13.82960	-3.14934	-0.84246

H	12.50249	-3.90076	0.08883
C	-2.05099	8.79320	0.10091
C	-2.28249	10.30121	0.11693
H	-2.29749	8.36551	1.07426
H	-2.66748	8.32429	-0.66965
O	-3.64894	10.50264	0.43013
H	-2.03939	10.74180	-0.86319
H	-1.63743	10.78417	0.86873
C	-3.99526	11.87749	0.47849
H	-3.41860	12.41204	1.24829
H	-5.05825	11.93154	0.72601
H	-3.82578	12.37116	-0.49026
H	-4.50773	1.05481	4.43574
C	-3.65438	0.59328	3.94701
H	-2.84624	2.45860	3.20928
C	-2.72665	1.38063	3.26076
H	-4.18657	-1.41060	4.54065
C	-3.47544	-0.78858	4.00479
C	-1.63625	0.78626	2.62938
C	-2.37785	-1.38709	3.38099
H	-0.92689	1.41708	2.10938
C	-1.44817	-0.60568	2.67847
H	-2.25540	-2.46010	3.45601
P	0.04502	-1.31211	1.86092
H	2.92424	-1.65547	1.58918

H	-1.79477	-3.54538	1.32377
C	2.76847	-0.95914	2.40343
C	1.46763	-0.57346	2.76970
C	3.87611	-0.47533	3.09923
H	4.86954	-0.79551	2.80213
C	-0.98745	-3.93125	1.93474
C	1.30353	0.30392	3.85368
C	0.06190	-3.09102	2.34833
C	3.70345	0.40869	4.16597
C	2.41584	0.79421	4.54067
H	0.31426	0.60443	4.17541
H	-1.84584	-5.89806	2.00074
C	-1.02346	-5.26891	2.32852
H	4.56625	0.78923	4.70523
H	2.26906	1.47327	5.37568
C	1.07045	-3.62814	3.16525
C	-0.00921	-5.79567	3.12985
H	1.88434	-3.00690	3.51751
C	1.03581	-4.97067	3.54655
H	-0.03565	-6.83929	3.43011
H	1.82724	-5.36536	4.17721

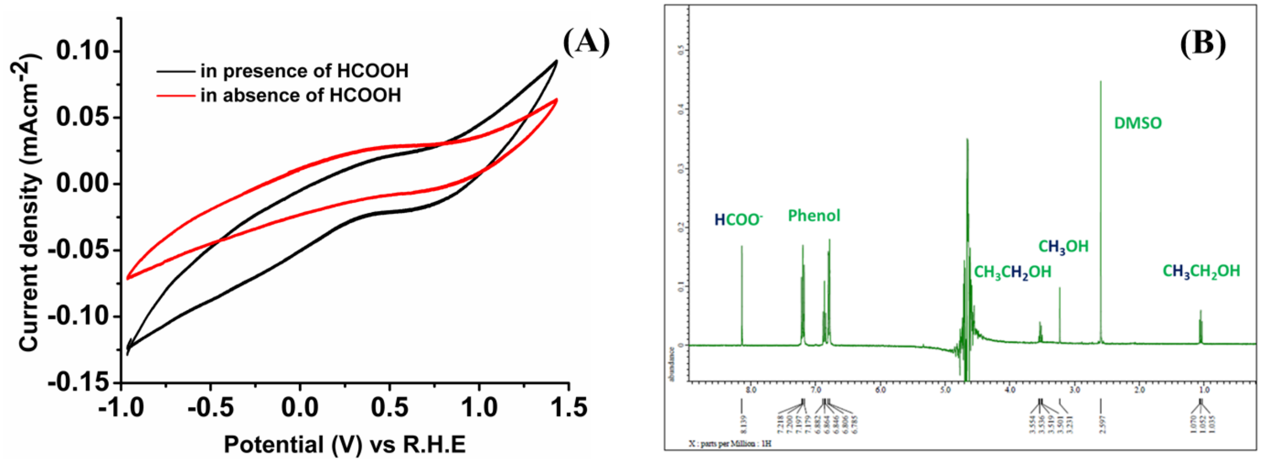

Supplementary Figure 34. 10 mM formic acid was added to 0.1 M $NaClO_4$ (pH = 6, 0.1 M phosphate buffer) and reduction studies were conducted at the potential -0.73 V vs RHE with Co-Corrole modified carbon paper electrodes. Cyclic voltammetry of Co-Corrole-carbon paper electrodes were measured (Supplementary Figure 34 A) which showed an increase in current density upon addition of 10 mM HCOOH. Reduction of formic acid yielded methanol and ethanol which was detected using GC-MS and ^{1}H-NMR spectra (Supplementary Figure 34 B). This brings us to the conclusion that formic acid is a key 2-electron reduced intermediate for the catalytic conversion of CO_2 to ethanol and methanol. (A) Cyclic voltammetry of Co-Corrole-carbon paper in 0.1 M $NaClO_4$ (pH = 6.0, 0.1 M Phosphate buffer) with 10 mM formic acid under Ar atmosphere (B) ^{1}H-NMR spectrum of the liquid products formed after the reduction of formic acid by Co-Corrole-carbon paper electrode after 4000 s (-0.73 V vs RHE) indicating methanol and ethanol formation.

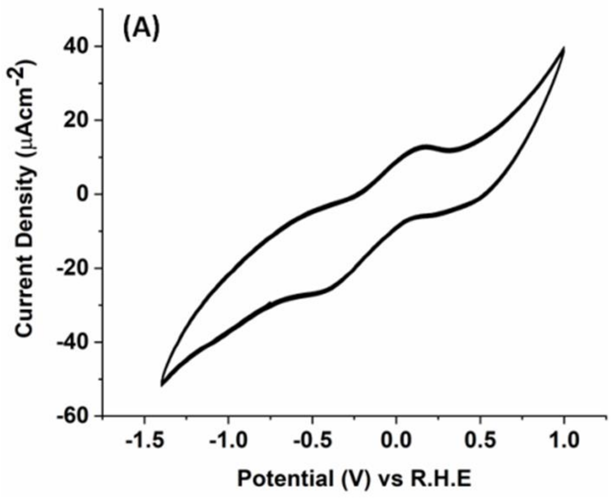

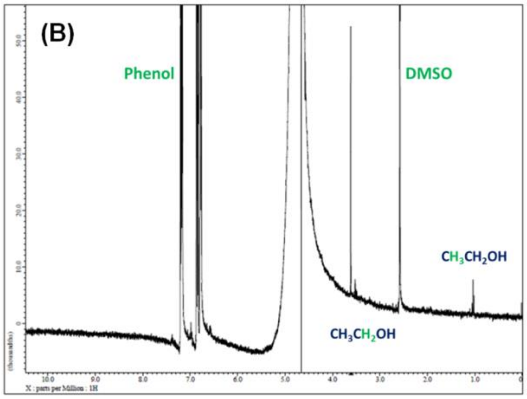

Supplementary Figure 35. 0.1 mM glyoxal was added to 0.1 M $NaClO_4$ (pH = 6, 0.1 M phosphate buffer) and reduction studies were conducted at the potentials -0.70 V to 0.80 V vs RHE with Co-Corrole modified carbon paper electrodes. Cyclic voltammetry of Co-Corrole-carbon paper electrodes were measured (Supplementary Figure 35A), which showed an increase in current density upon addition of 0.1 mM glyoxal. Reduction of glyoxal yielded ethanol (Supplementary Figure 35B) which brings us to the conclusion that the glyoxal is a key intermediate for the catalytic conversion of CO_2 to ethanol. Similar experiments were conducted with 0.1 mM formaldehyde and 0.1 mM methanol under the same reaction conditions which showed no transformation. (A) Cyclic voltammetry of Co-Corrole-carbon paper in 0.1 M $NaClO_4$ (0.1 M pH = 6.0 phosphate buffer) with 0.1 mM glyoxal under Ar atmosphere (B) ^{1}H-NMR spectrum of the liquid products formed after the reduction of glyoxal by Co-Corrole-carbon paper electrode (-0.73 V vs RHE). The ^{1}H- NMR signal (singlet) at 3.65 ppm reflects the formation of ethylene glycol.

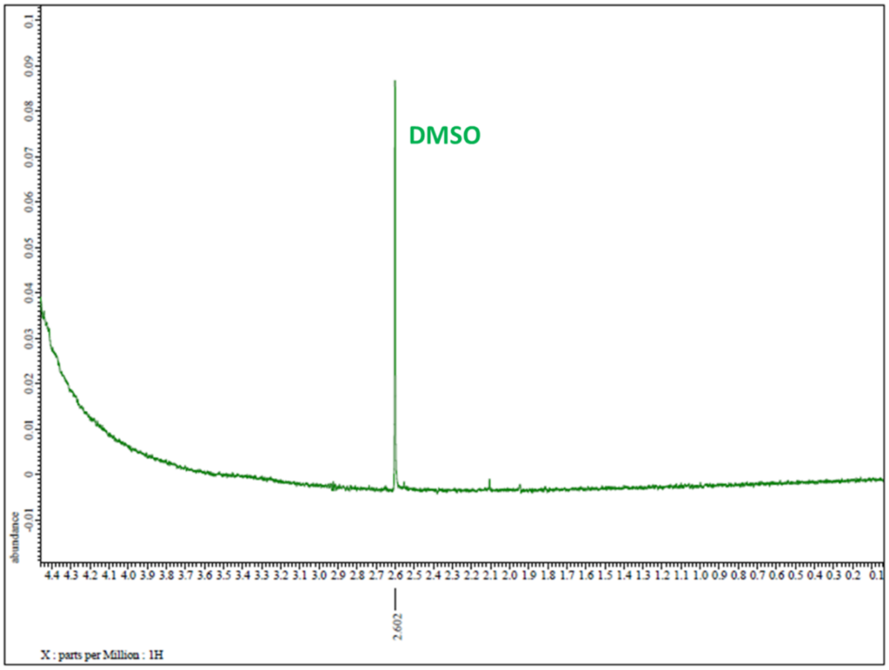

Supplementary Figure 36. ^{1}H-NMR spectrum of the electrolyte after 4000 s of CO_2 electrolysis over unmodified carbon paper electrode at -0.73 V vs RHE.

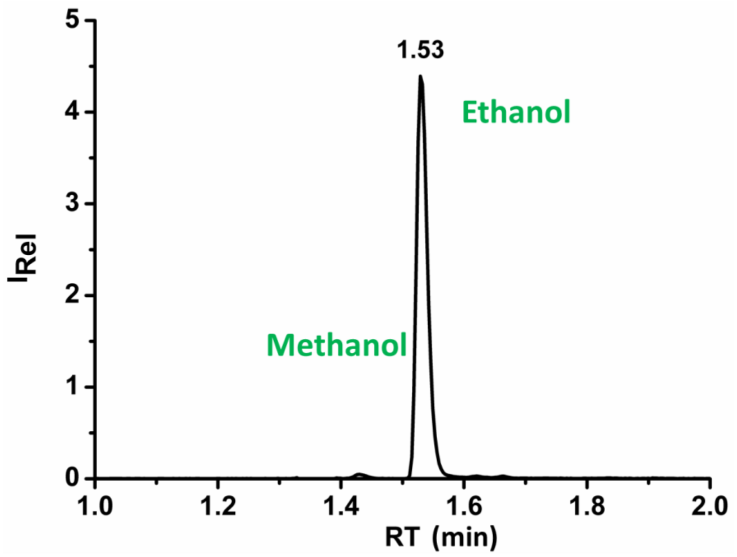

Supplementary Figure 37. GC-MS retention time of the liquid products after CO_2 electrolysis for 4000 s at -0.8 V vs RHE showing methanol at 1.41 and ethanol at 1.53 min of retention times.

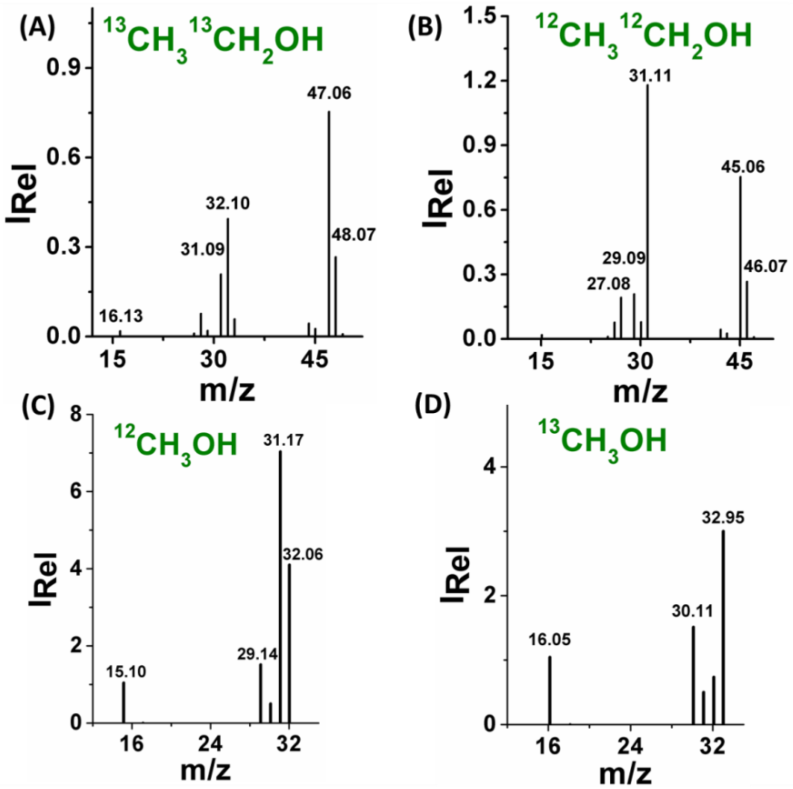

Supplementary Figure 38. Obtained mass spectrum at the above shown GC retention times 1.53 min for ethanol: (A) ^{13}C enriched, (B) ^{12}C enriched and 1.41 min for methanol: (C) ^{12}C enriched, (D) ^{13}C enriched.

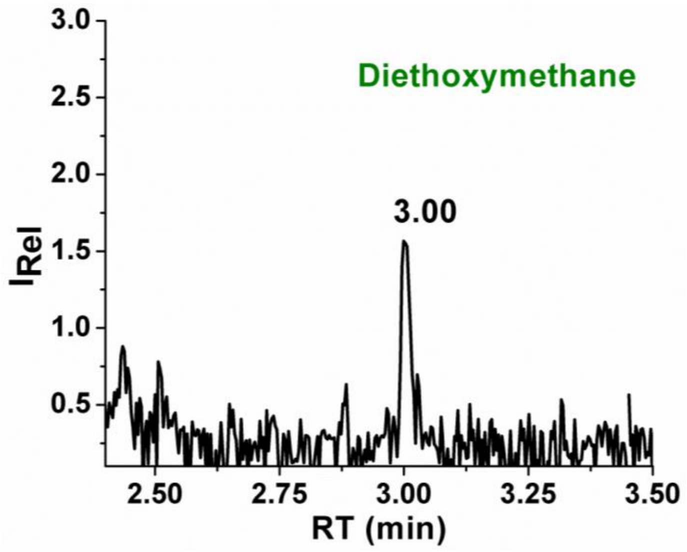

Supplementary Figure 39. GC-MS of diethoxymethane after CO_2 electrolysis at -0.8 V vs RHE with a retention time of 3.00 min.

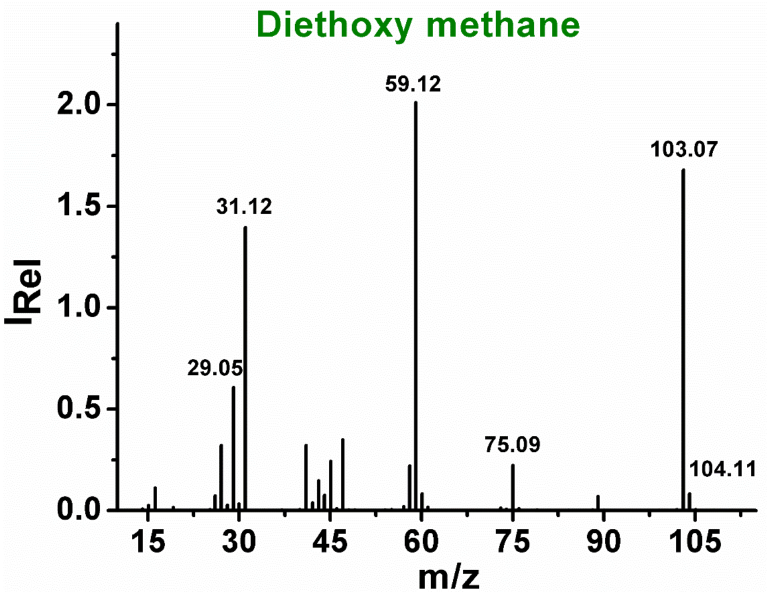

Supplementary Figure 40. Obtained mass spectrum of the above shown GC retention time 3.00 min.

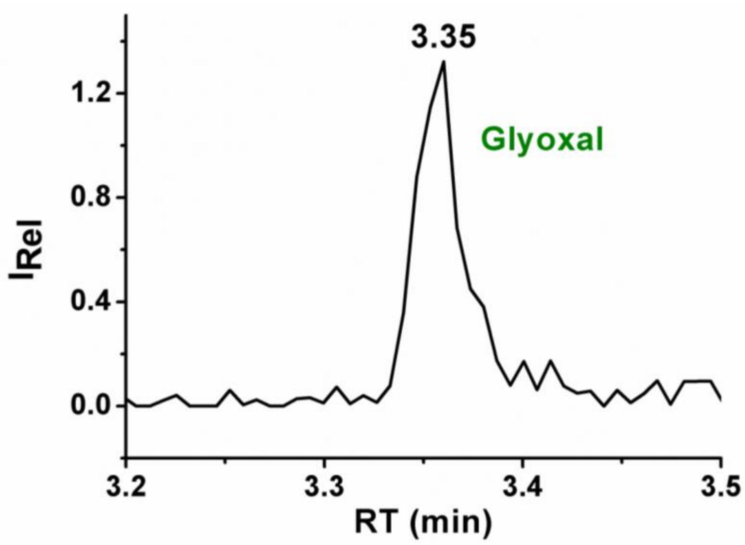

Supplementary Figure 41. GC-MS of glyoxal after CO_2 electrolysis at -0.8 V vs RHE with a retention time of 3.35 min.

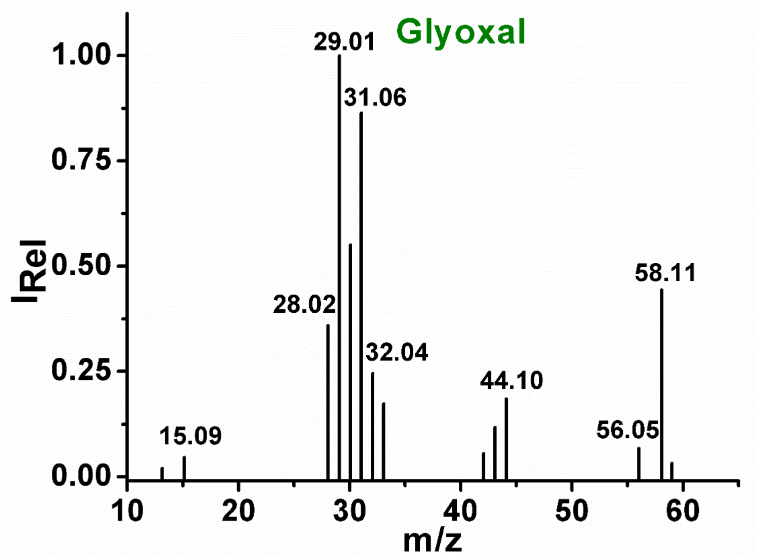

Supplementary Figure 42. Obtained mass spectrum of the above shown GC retention time 3.35 min.

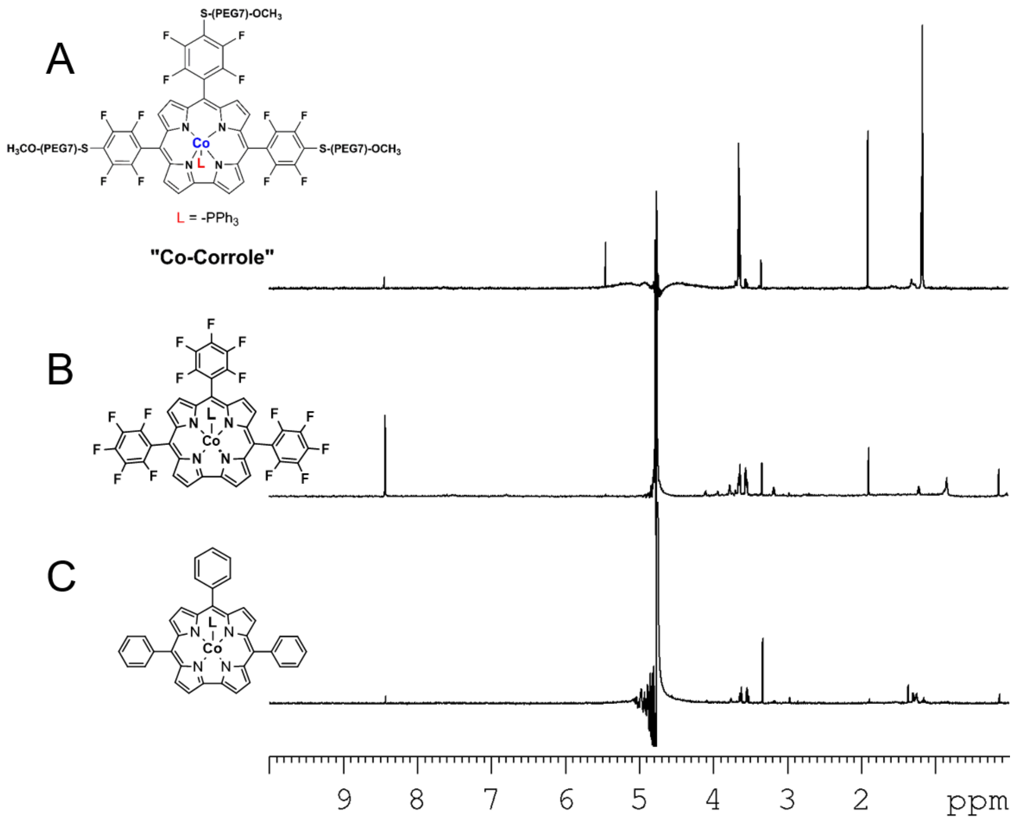

Supplementary Figure 43. ^{1}H-NMR analyses after the electroreduction reaction with A) Co-Corrole, B) PPh_3-CoTpFPC, and C) PPh_3-CoTPC catalysts on carbon fiber electrode.

References

1. Hahn, C. *et al.* Engineering Cu surfaces for the electrocatalytic conversion of CO2. Controlling selectivity toward oxygenates and hydrocarbons. *Proc. Natl. Acad. Sci. U.S.A.,* 201618935 (2017).

2. Ren, D., Ang, B. S.-H. & Yeo, B. S. Tuning the selectivity of carbon dioxide electroreduction toward ethanol on oxide-derived Cu x Zn catalysts. *ACS Catal.* **6,** 8239–8247 (2016).

3. Kim, D., Kley, C. S., Li, Y. & Yang, P. Copper nanoparticle ensembles for selective electroreduction of CO2 to C2–C3 products. *Proc. Natl. Acad. Sci. U.S.A.* **114,** 10560–10565 (2017).

4. Liu, Y. *et al.* Selective Electrochemical Reduction of Carbon Dioxide to Ethanol on a Boron-and Nitrogen-Co-doped Nanodiamond. *Angew. Chem. Int. Ed.* **56,** 15607–15611 (2017).

5. Albo, J. & Irabien, A. Cu 2 O-loaded gas diffusion electrodes for the continuous electrochemical reduction of CO 2 to methanol. *Journal of Catalysis* **343,** 232–239; 10.1016/j.jcat.2015.11.014 (2016).

6. Handoko, A. D. *et al.* Mechanistic insights into the selective electroreduction of carbon dioxide to ethylene on Cu2O-derived copper catalysts. *J. Phys. Chem. C* **120,** 20058–20067 (2016).

7. Albo, J. *et al.* Copper-Based Metal-Organic Porous Materials for CO2 Electrocatalytic Reduction to Alcohols. *ChemSusChem* **10,** 1100–1109; 10.1002/cssc.201600693 (2017).

8. Loiudice, A. *et al.* Tailoring copper nanocrystals towards C2 products in electrochemical CO2 reduction. *Angew. Chem. Int. Ed.* **55,** 5789–5792 (2016).

9. Ren, D. *et al.* Selective electrochemical reduction of carbon dioxide to ethylene and ethanol on copper (I) oxide catalysts. *ACS Catal.* **5,** 2814–2821 (2015).

10. Hori, Y., Takahashi, I., Koga, O. & Hoshi, N. Electrochemical reduction of carbon dioxide at various series of copper single crystal electrodes. *Journal of Molecular Catalysis A: Chemical* **199,** 39–47; 10.1016/S1381-1169(03)00016-5 (2003).

11. Lin, S. *et al.* Covalent organic frameworks comprising cobalt porphyrins for catalytic CO_2 reduction in water. *Science* **349,** 1208–1213; 10.1126/science.aac8343 (2015).

12. Dunwell, M. *et al.* The Central Role of Bicarbonate in the Electrochemical Reduction of Carbon Dioxide on Gold. *Journal of the American Chemical Society* **139,** 3774–3783; 10.1021/jacs.6b13287 (2017).

13. Shen, J. *et al.* Electrocatalytic reduction of carbon dioxide to carbon monoxide and methane at an immobilized cobalt protoporphyrin. *Nat. Commun.* **6,** 8177 (2015).

14. Mondal, A., Adhikary, B. & Mukherjee, D. Room-temperature synthesis of air stable cobalt nanoparticles and their use as catalyst for methyl orange dye degradation. *Colloids and Surfaces A: Physicochemical and Engineering Aspects* **482,** 248–257; 10.1016/j.colsurfa.2015.05.011 (2015).

15. Chai, J.-D. & Head-Gordon, M. Long-range corrected hybrid density functionals with damped atom–atom dispersion corrections. *Phys. Chem. Chem. Phys.* **10,** 6615–6620 (2008).

16. Grimme, S. Accurate description of van der Waals complexes by density functional theory including empirical corrections. *J. Comput. Chem.* **25,** 1463–1473 (2004).

17. M. J. Frisch, G. W. Trucks, H. B. Schlegel, G. E. Scuseria, M. A. Robb, J. R. Cheeseman, G. Scalmani, V. Barone, B. Mennucci, G. A. Petersson, H. Nakatsuji, M. Caricato, X. Li, H. P. Hratchian, A. F. Izmaylov, J. Bloino, G. Zheng, J. L. Sonnenberg, M. Hada, M. Ehara, K. Toyota, R. Fukuda, J. Hasegawa, M. Ishida, T. Nakajima, Y. Honda, O. Kitao, H. Nakai, T. Vreven, J. A. Montgomery, Jr., J. E. Peralta, F. Ogliaro, M. Bearpark, J. J. Heyd, E. Brothers, K. N. Kudin, V. N. Staroverov, R. Kobayashi, J. Normand, K. Raghavachari, A. Rendell, J. C. Burant, S. S. Iyengar, J. Tomasi, M. Cossi, N. Rega, J. M. Millam, M. Klene, J. E. Knox, J. B. Cross, V. Bakken, C. Adamo, J. Jaramillo, R. Gomperts, R. E. Stratmann, O. Yazyev, A. J. Austin, R. Cammi, C. Pomelli, J. W. Ochterski, R. L. Martin, K. Morokuma, V. G. Zakrzewski, G. A. Voth, P. Salvador, J. J. Dannenberg, S. Dapprich, A. D. Daniels, O. Farkas, J. B. Foresman, J. V. Ortiz, J. Cioslowski, D. J. Fox. *Gaussian 09, Revision D.* (Gaussian, Inc., Wallingford CT, 2013).

18. Wong, M. W., Gill, P. M. W., Nobes, R. H. & Radom, L. 6-311G (MC)(d, p): a second-row analogue of the 6-311G (d, p) basis set: calculated heats of formation for second-row hydrides. *J. Phys. Chem.* **92,** 4875–4880 (1988).

19. Cances, E., Mennucci, B. & Tomasi, J. A new integral equation formalism for the polarizable continuum model. Theoretical background and applications to isotropic and anisotropic dielectrics. *J. Chem. Phys.* **107,** 3032–3041 (1997).

20. Cossi, M., Scalmani, G., Rega, N. & Barone, V. New developments in the polarizable continuum model for quantum mechanical and classical calculations on molecules in solution. *J. Chem. Phys.* **117,** 43–54 (2002).

21. Toda, Y. *et al.* Activation and splitting of carbon dioxide on the surface of an inorganic electride material. *Nature Communications* **4,** 2378; 10.1038/ncomms3378 (2013).

22. Nielsen, I. M. B. & Leung, K. Cobalt-porphyrin catalyzed electrochemical reduction of carbon dioxide in water. 1. A density functional study of intermediates. *The journal of physical chemistry. A* **114,** 10166–10173; 10.1021/jp101180m (2010).

23. Shen, J., Kolb, M. J., Göttle, A. J. & Koper, M. T. M. DFT Study on the Mechanism of the Electrochemical Reduction of CO 2 Catalyzed by Cobalt Porphyrins. *The Journal of Physical Chemistry C* **120,** 15714–15721; 10.1021/acs.jpcc.5b10763 (2016).

24. Isse, A. A. & Gennaro, A. Absolute potential of the standard hydrogen electrode and the problem of interconversion of potentials in different solvents. *The journal of physical chemistry. B* **114,** 7894–7899; 10.1021/jp100402x (2010).
